# Genomic Reconstruction of the Successful Establishment of a Feralized Bovine Population on the Subantarctic Island of Amsterdam

**DOI:** 10.1093/molbev/msae121

**Published:** 2024-06-18

**Authors:** Mathieu Gautier, Thierry Micol, Louise Camus, Katayoun Moazami-Goudarzi, Michel Naves, Elise Guéret, Stefan Engelen, Arnaud Lemainque, François Colas, Laurence Flori, Tom Druet

**Affiliations:** CBGP, INRAE, CIRAD, IRD, L’institut Agro, Université de Montpellier, Montpellier, France; LPO, Rochefort, France; CBGP, INRAE, CIRAD, IRD, L’institut Agro, Université de Montpellier, Montpellier, France; GABI, INRAE, AgroParisTech, Université Paris-Saclay, Jouy-en-Josas, France; ASSET, INRAE, Petit-Bourg, Guadeloupe, France; MGX-Montpellier GenomiX, University of Montpellier, CNRS, INSERM, Montpellier, France; Retired, CEA, Institut de biologie François-Jacob, Genoscope, Université Paris-Saclay, Evry, France; Retired, CEA, Institut de biologie François-Jacob, Genoscope, Université Paris-Saclay, Evry, France; Retired, Saint-Paul and Amsterdam District, Terres Australes et Antarctiques Françaises, France; SELMET, INRAE, CIRAD, L’institut Agro, Université de Montpellier, Montpellier, France; Unit of Animal Genomics, GIGA-R and Faculty of Veterinary Medicine, University of Liège, Liège, Belgium

**Keywords:** cattle, Amsterdam island, demographic inference, genetic load, feralization, adaptation

## Abstract

The feral cattle of the subantarctic island of Amsterdam provide an outstanding case study of a large mammalian population that was established by a handful of founders and thrived within a few generations in a seemingly inhospitable environment. Here, we investigated the genetic history and composition of this population using genotyping and sequencing data. Our inference showed an intense but brief founding bottleneck around the late 19th century and revealed contributions from European taurine and Indian Ocean Zebu in the founder ancestry. Comparative analysis of whole-genome sequences further revealed a moderate reduction in genetic diversity despite high levels of inbreeding. The brief and intense bottleneck was associated with high levels of drift, a flattening of the site frequency spectrum and a slight relaxation of purifying selection on mildly deleterious variants. Unlike some populations that have experienced prolonged reductions in effective population size, we did not observe any significant purging of highly deleterious variants. Interestingly, the population’s success in the harsh environment can be attributed to preadaptation from their European taurine ancestry, suggesting no strong bioclimatic challenge, and also contradicting evidence for insular dwarfism. Genome scan for footprints of selection uncovered a majority of candidate genes related to nervous system function, likely reflecting rapid feralization driven by behavioral changes and complex social restructuring. The Amsterdam Island cattle offers valuable insights into rapid population establishment, feralization, and genetic adaptation in challenging environments. It also sheds light on the unique genetic legacies of feral populations, raising ethical questions according to conservation efforts.

## Introduction

Understanding the successful establishment of introduced species in novel environments is a fundamental area of ecological and evolutionary research. Criteria for a population to be deemed “established” involve its capacity for self-sustained reproduction without additional introductions (Sol 2007). The success of the establishment is contingent upon various factors, including the adaptability of nonnative species to the new environment. For example, fast life histories, characterized by frequent reproduction, high fecundity, and early maturity, are often perceived as reducing the risk of extinction for newly introduced species (Capellini et al. 2015). However, in several mammalian species, the relationship between fast life histories and establishment success has proven inconsistent (Sol et al. 2008; Capellini et al. 2015). On the contrary, “propagule pressure,” encompassing both the number of introduced individuals (propagule size) and their arrival patterns (number and spatiotemporal distribution of introduction events), emerges as a critical factor in successful establishment (Simberloff 2009).

Both empirical and theoretical evidence support a positive correlation between propagule pressure and establishment probability (Lockwood et al. 2005; Simberloff 2009), especially within the range of 10 to 100 introduced individuals (Cassey et al. 2018). From a genetic diversity perspective, low propagule pressure can lead to increased inbreeding and genetic drift, resulting in high genetic load and reduced fitness of offspring (Keller and Waller 2002). Consequently, it is generally challenging for a small number of individuals (<10) to establish in a nonnative habitat (Cassey et al. 2018). In contrast, higher propagule pressure, which typically involves the introduction of more individuals with potentially more diverse genetic backgrounds, increases genetic variation within the founding population, which can then adapt to a wider range of conditions and increase the population’s chances of survival.

Yet, this conventional view of the importance of genetic diversity is challenged by the “genetic paradox of invasion.” This paradox arises from numerous cases in which nonnative species become invasive with only a small founding population (Estoup et al. 2016). It can be resolved if there is no substantial genetic impoverishment in the invasive populations compared to the native ones, or if the founding individuals were preadapted to the new environment, as in the scenario known as “anthropogenetically induced adaptation to invade” or AIAI (Hufbauer et al. 2012; Estoup et al. 2016). In some cases, the paradox could also be explained by an overall increase in population fitness resulting from the purging of genetic load, which is reportedly more effective for bottlenecks of intermediate intensity (e.g. moderate reductions in population size over multiple generations) and for the removal of recessive and highly deleterious variants (Glémin 2003; Estoup et al. 2016).

The cattle population of the subantarctic island of Amsterdam provides a remarkable opportunity to study the successful establishment of a large mammal population from an extremely low propagule pressure in a new seemingly inhospitable environment. Historical records (Lesel 1969) indicate that this population was initially established by just five animals introduced by a French farmer from La Réunion island in the Indian Ocean and subsequently abandoned on the remote island of Amsterdam in 1871. This island, measuring 55 km^2^ and located 4,440 km southeast of Madagascar, is now part of the Terres Australes et Antarctiques Françaises (TAAF or TAF). Since their introduction, the cattle have been free to roam and breed, with the population reaching peaks of around 2,000 individuals in 1952 and 1988 (Micol and Jouventin 1995), making it one of the few known feral cattle populations (Hall 2004). Due to concerns about the threat posed by their proliferation to endemic species, control measures aimed at restoring the island’s terrestrial ecosystem were implemented from 1988 (Micol and Jouventin 1995), before the cattle population was eradicated in 2010 following a decision by the authorities but without any effort to collect biological samples. Fortunately, unique DNA samples from 18 individuals, obtained during two previous sampling campaigns in 1992 and 2006, were sufficiently preserved to allow for genotyping and whole-genome sequencing (WGS). This enabled us to generate new genetic data for a comprehensive genomic characterization of the Amsterdam Island cattle population, henceforth referred to as the TAF population.

This study aims to gain insight into the genetic bases that enabled the successful establishment and feralization of a large domestic mammal from an extreme but short population bottleneck. Our analysis first includes a detailed inference of demographic history based on genetic data. Second, we relied on WGS data to assess levels of genetic diversity and genetic load, thereby searching for evidence of relaxation of purifying selection. Finally, we jointly used WGS and genotyping data to characterize the adaptive history of the TAF population, including (i) an assessment of the genetic maladaptation (Capblancq et al. 2020) of domestic cattle to the Amsterdam island environment; (ii) the critical evaluation of the island dwarfism hypothesis as recently proposed based on crude phenotypic data (Rozzi and Lomolino 2017); and (iii) the identification of key physiological functions mobilized by selection from the genes surrounding our identified genomic footprints of selection.

## Results

### The Genetic History of the Cattle of the Amsterdam Island

**Fig. 1.**
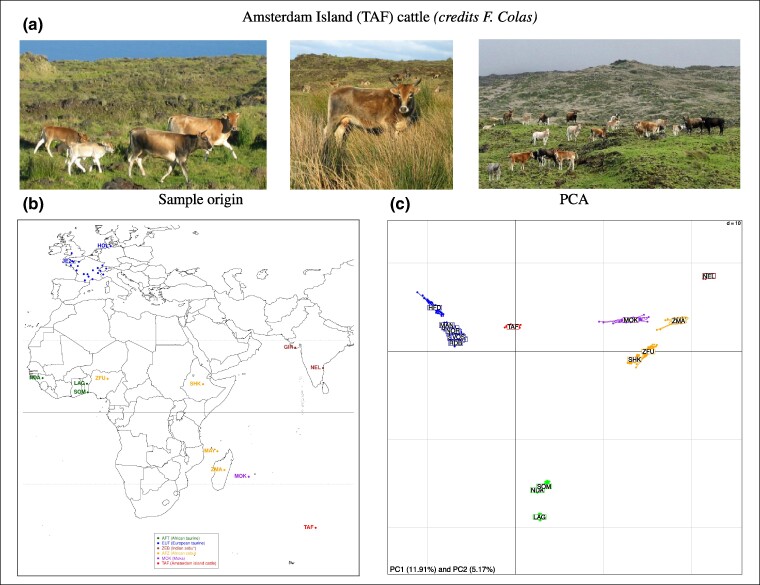
a) Pictures of cattle from the island of Amsterdam island (credits: François Colas). b) Location map of cattle breeds included in the W50K data set. c) Results of the PCA of the W50K data set. Individuals are plotted on the first two principal components according to their coordinates. Ellipses characterize the dispersion of each breed around its centroid.

A total of 18 TAF individuals (Fig. 1a) sampled in 1992 (n=12) and 2006 (n=6) during two different campaigns (supplementary table S1, Supplementary Material online) were newly genotyped on the commercial BovineSNP50K assay (Matukumalli et al. 2009) along with 31 individuals from the Moka Zebu (MOK) population, a local breed living on the La Réunion island that was considered a possible proxy for the TAF founders. These data were combined with public data from 30 other cattle populations representing the worldwide cattle diversity including, (i) two Brazilian Zebu breeds originating from India (ZEB); (ii) three African taurine (AFT) breeds; (iii) four African Zebu breeds (AFZ) including the two closely related Indian Ocean Zebu breeds from the islands of Mayotte (MAY) and Madagascar (Magnier et al. 2022); and (iv) 21 European (mostly French) taurine breeds (EUT) (supplementary table S2, Supplementary Material online, Fig. 1b). After filtering, the final combined data set (hereafter referred to as W50K), consisted of 876 individuals from 32 different populations genotyped at 40,426 autosomal single nucleotide polymorphisms (SNPs) (supplementary table S2, Supplementary Material online).

#### Genetic Structuring of the TAF Population

As detailed in supplementary table S3, Supplementary Material online, 43.3% of these SNPs were monomorphic in the TAF sample, which was the highest level among observed values ranging from 39.6% to 40.8% in ZEB; 23.5% to 41.0% in AFT; 19.5% to 34.0% in AFZ; and 7.41% to 21.5% in EUT. Due to the strong SNP ascertainment bias toward SNPs of EUT origin (Matukumalli et al. 2009; Gautier et al. 2010), the within-population genetic diversity estimates obtained from the BovineSNP50K assay data must be interpreted with great caution, particularly for the ZEB, AFZ, and AFT breeds (see below for accurate estimates based on WGS data). Nevertheless, the estimated heterozygosity of 0.216 observed in TAF was lower than that observed in EUT (from 0.258 in RDB to 0.323 in HFD) and in the range of that observed in AFZ populations (from 0.204 in ZMA to 0.255 in SHK). More interestingly, the $F_{\mathrm{IS}}$ estimated within the TAF sample was found to be significantly negative indicating an excess of heterozygotes in the sample relative to the Hardy–Weinberg equilibrium expectation, although the 18 samples from the 1992 and 2006 campaigns were analyzed together. In addition, the estimated pairwise kinship coefficients among the 12 samples collected in 1992 (Manichaikul et al. 2010) ranged from −0.039 to 0.26 (median of 0.014), suggesting a few close parental relationships (supplementary fig. S1, Supplementary Material online). For example, the relationship between TAF_5609 (born 1985) and TAF_5608 (born 1989) was estimated to be within [0.177,0.354], suggesting a first-degree relationship corresponding to a mother-daughter relationship according to the sharing of IBD (Identical by descent) segments. The four coefficients between TAF_5608 and TAF_5612 (ϕ=0.144); TAF_5610 and TAF_5617 (ϕ=0.131); TAF_5609 and TAF_5619 (ϕ=0.101); and TAF_5608 and TAF_5610 (ϕ=0.0887) were within [0.0884,0.177], suggesting a second-degree relationship. Analysis of IBD-segment sharing allowed the relationship between TAF_5608 and TAF_5612 to be upgraded to a full-sib relationship, birth dates suggesting half sibs or aunt-niece relationships for the other three pairs. Note that at least four of these six females were observed to belong to the same group of about 40 individuals during the sampling campaign (Micol, personal observation). The remaining 61 coefficients between the 1992 individuals were all <0.0884 (relationship more distant than second degree). Similarly, among the 15 pairs of 2006 samples, the estimated kinship coefficients were <0.0884 ranging from −0.035 to 0.074 (median of 0.013), as were the 72 pairwise comparisons between individuals collected in 1992 and 2006 which ranged from −0.091 to 0.062 (median of −0.0081) and were shifted toward lower values, as expected. To assess the impact of these few pairs of related individuals, we repeated all subsequent analyses based on the W50K data set using only the ten individuals deemed as unrelated up to third-degree relationship (supplementary fig. S1, Supplementary Material online) and obtained results in very good agreement to those presented (data not shown).

#### Relationship with Other Worldwide Populations

The global $F_{\mathrm{ST}}$ estimated on the W50K data set was found to be 21.5% (95%CI=[21.1%;21.8%]) while the global $F_{\mathrm{IS}}$ remained negligible (95%CI=[0.04%;0.26%]). As shown in supplementary fig. S2, Supplementary Material online, the estimated pairwise $F_{\mathrm{ST}}$ values ranged from 0.0247 for the MAY/ZMA pair to 0.472 for the LAG/NEL pair (median of 0.203). For pairs including the TAF, values ranged from 0.208 (TAF/PMT) to 0.436 (TAF/NEL), consistent with a presumably high amount of drift in this population following an intense founding bottleneck. In addition, the TAF appeared more closely related to breeds of EUT origin. However, clustering based on pairwise $F_{\mathrm{ST}}$ must be interpreted cautiously and cannot be safely used to infer population origins as it is highly sensitive to drift and admixture. For example, the TAF may appear as an outgroup of the EUT cluster in supplementary fig. S2, Supplementary Material online due to drift and the TAF/PMT pair is likely to display the lowest pairwise $F_{\mathrm{ST}}$ because, like the TAF, the PMT breed has some amount of ZEB ancestry (see supplementary fig. S3, Supplementary Material online and Gautier et al. 2010). Finally, note that the $F_{\mathrm{ST}}$ between the TAF and the newly genotyped MOK sample was 0.275, i.e. in the upper range of values estimated for pairs involving this population sample, which varied from 0.0578 for the MOK/ZMA pair to 0.303 for the MOK/LAG pair.

To refine the exploration of the overall structuring of genetic diversity on the W50K data set, we computed allele-sharing distances (ASDs) between all pairs of individuals and used the resulting matrix to build the neighbor-joining tree shown in supplementary fig. S4, Supplementary Material online. In agreement with previous observations (e.g. Gautier et al. 2010) and the results above, the obtained tree showed a clear clustering of individuals according to their population of origin, except to some extent for the MOK individuals, which were more dispersed with two apparent outliers. At a higher level, the EUT, AFT, and ZEB populations were also well separated, as were the AFZ and MOK populations, both of which branched between ZEB and AFT. Finally, the TAF population was clearly isolated from the other populations and branched close to the EUT populations. A similar pattern was recovered using principal component analysis (PCA) of the W50K data set (Fig. 1c). Interestingly, in the first factorial plan, TAF and MOK individuals are positioned on an axis connecting the ZMA and EUT populations, suggesting ZEB and EUT ancestry for both populations, with TAF individuals closer to EUT and MOK closer to ZMA and MAY. An alternative but qualitatively similar representation was obtained using unsupervised hierarchical clustering (supplementary fig. S3, Supplementary Material online). For example, at K=3 predefined clusters, the proportion of the “green” cluster (interpreted as EUT) ranged from 68.0% to 75.0% (median of 71.4%) for TAF individuals and from 19.8% to 26.4% (median of 23.0%) for MOK individuals. However, the exact origins of the different ancestries remain difficult to assess with such exploratory analyses and should, therefore, be interpreted with caution.

#### Inferring the Demographic History of the TAF Population

We relied on the *f*-statistics based framework (Patterson et al. 2012) as implemented in the R package *poolfstat* (Gautier et al. 2022) to infer the origin of the TAF population using the W50K data set. We first performed a formal test of admixture based on the $F_{3}$ statistic computed for all the 465 population triplets with TAF as the target admixed population, but none of the resulting statistics were found to be (significantly) negative. In fact, this can be explained by the high amount of drift due to the extreme founding effect in the TAF history. Conversely, 100 of the 465 population triplets with MOK as the target population showed significantly negative $\;f_{3}$ (at the 5% threshold), with the lowest value observed for the (MOK;GIR,NDA) and (MOK;BPN,ZMA) configurations (both with Z=−21.7).

**Fig. 2.**
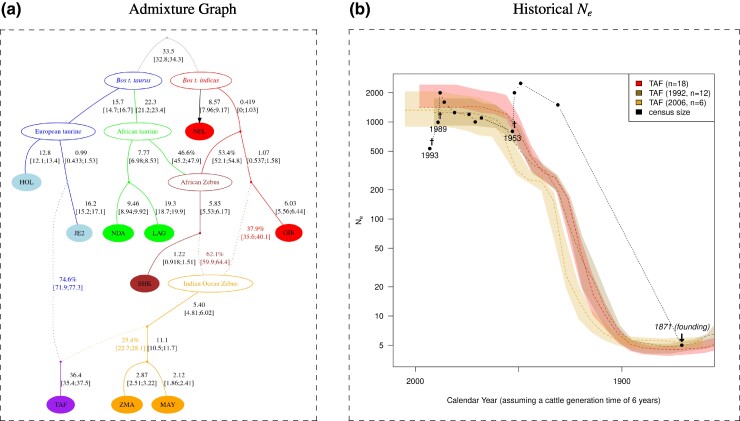
The demographic history of the Amsterdam island cattle (TAF) population inferred from genotyping data (W50K data set). a) Admixture graph linking the TAF population with Indian Ocean Zebu (MAY and ZMA), Zebu breeds of Indian origin (GIR and NEL), AFT (NDA and LAG) and European taurine breeds (HOL and JE2) inferred with *poolfstat* (Gautier et al. 2022). Admixture events are indicated by dotted arrows. Estimates of branch lengths in drift units (×100) and admixture rates are given next to the corresponding edges with (approximate) blockjackknife 95% CI in brackets. This is the graph with the best fit (BIC 3.4 units lower than the graph with the second lowest BIC) among all possible ways of positioning TAF and JE2 on a scaffold graph reproducing the graph obtained by Magnier et al. (2022) to infer the history of Indian Ocean island populations. The *Z*-score for the worst fitting *f*-statistics, $f_{4}(\mathrm{HOL},\mathrm{JE}2;\mathrm{MAY},\mathrm{NDA})$ is −2.02. b) Recent population size history ($N_{e}$) estimated with the program GONE (Santiago et al. 2020) for the TAF population analyzing the two samples collected in 1992 and 2006 separately and together. For each samples (with size, collection date and color code indicated in the legend box), the average $N_{e}$ trajectories (dashed line) and 95% confidence envelope estimated from blockjackknife sampling are plotted. The time scale has been transformed to calendar years assuming a 6-year generation time for cattle and accounting for each collection date. Census sizes obtained from historical records (see Table 2 in Micol and Jouventin 1995) are overlaid with black dots. The year of introduction of cattle to the island (1871) and the three (known) major events that led to sharp population declines in 1953 after an infectious disease outbreak (probably paratuberculosis, Lesel 1969) and in 1988 and 1993 after regulation culling are highlighted on the plot.

We also used *f*-statistics to construct an admixture graph as detailed in supplementary fig. S5, Supplementary Material online (Patterson et al. 2012; Gautier et al. 2022). This allowed us to demonstrate the admixed origin of the TAF and MOK populations with closely related EUT ancestral sources contributing to 75% and 22% of their genome composition, respectively, and another source closely related to ZMA. Positioning the MOK population resulted in a poorer fit suggesting a more complex origin (i.e. three-way EUT×ZMA×ZEB admixture) difficult to capture with the W50K data set (supplementary fig. S5, Supplementary Material online). Nevertheless, the inferred graph suggests that MOK may not be considered as the closest proxy for the source populations from which TAF originated. Figure 2a shows the results for the best-fitting graph among all possible ways of positioning TAF and the JE2 Jersiaise breed sample (supplementary table S2, Supplementary Material online) on a scaffold graph previously inferred to describe the history of the Indian Ocean island populations MAY and ZMA (Magnier et al. 2022). This graph included, in addition to MAY and ZMA, two ZEB (GIR and NEL), two AFT (LAG and NDA), an East African Zebu (here replaced by the closely related SHK), and an EUT breed (HOL). The graph was actually very similar to that of supplementary fig. S5b, Supplementary Material online. Note that we chose to consider JE2 because it was the EUT breed the most divergent to HOL (supplementary fig. S3, Supplementary Material online with K=4) and provided a very similar admixture graph than the one obtained using the JER sample but with a slightly better fit (not shown). With the addition of MAY, the ancestral TAF source related to Indian Ocean Zebu branches before the separation of MAY and ZMA, which we previously estimated to have started around the 16th century (Magnier et al. 2022). This result suggests that the population contributing to the Indian Ocean Zebu source was established early on the island of La Réunion. Alternatively, more recent exchanges between MAY and ZMA (Magnier et al. 2022) may have contributed to their closer proximity on the inferred graph. To provide insight into the EUT origin of the TAF (see supplementary fig. S6, Supplementary Material online), we ranked the 22 sampled EUT breeds by their proximity to the EUT ancestral source of the TAF using (i) the $\;f_{3}$ estimates for all the (TAF;ZMA,EUT) population triplet configurations (ranked in ascending order, supplementary table S5, Supplementary Material online); and (ii) the $\;f_{4}$ estimates for all the (EUT,NDA;TAF,GIR) population quadruplet configurations (ranked in decreasing order, supplementary table S6, Supplementary Material online). Although all confidence intervals (CIs) overlap, the closest breeds originate from northwestern Europe, in particular the island of Jersey. Based on the inferred admixture graph, we could obtain a more accurate estimate of the 95% CI for the admixture proportions using $F_{4}$-ratio (supplementary fig. S6, Supplementary Material online) giving 73.1% (95%CI=[70.7;77.3]) of EUT ancestry and 26.9% (95%CI=[22.7;29.3]) of Indian Ocean Zebu ancestry in the TAF population (consistent with Fig. 2a). Interestingly, the eight sequenced TAF individuals (see below) all carried the mitochondrial haplogroup “T1b1” found in most African cattle, including African Zebus (Ward et al. 2022). This same haplogroup was also present in six of the eight ZMA and one of the eight MAY individuals sequenced, with the remaining two ZMA and five MAY individuals carrying the closely related “T1b1b1a1” haplogroup (two MAY individuals carried “T1d”). This suggests that the TAF founders were mixed with local breeds before being moved to the island of Amsterdam.

#### Timing of Admixture of the TAF Source Population

We further estimated the timing of this admixture event using the ALDER program, which is based on modeling the exponential decay of admixture-induced linkage disequilibrium (LD) with genetic distance (Loh et al. 2013). Using TAF as the target admixed population in one-reference tests (i.e. using an LD measure weighted by allele frequencies [AFs] observed in a single source population proxy), a significant weighted LD curve was found with all the 31 other breeds of the W50K data set, except MOK and ZFU, confirming the admixture signal. We further fitted the TAF two-reference weighted LD curves (i.e. using an LD measure weighted by the AFs observed in two source proxies) for all 406 possible pairs of the 29 populations that passed the one-reference tests. A total of 73 tests were considered successful (i.e. gave parameter estimates consistent with the one-reference fitted curve obtained with either of the two reference populations). The highest amplitude (i.e. *y*-intercept) estimate was obtained with NEL and JE2 as the source proxies suggesting that these populations were the best closest proxy to the actual source populations (among those sampled that passed the tests). The corresponding estimated time for admixture was found to be $t_{a}=22.02\pm 2.50$ generations (i.e. year 1860±15 assuming a 6-year generation time and 1992 as the average birth year) and was consistent across all tests. Note that all two-reference weighted LD curves with ZMA (or MAY) and an EUT breed gave similar results, but the best-fitting estimates of the timing of admixture were discordant between those obtained with fitting the decay of LD weighted with the two or either one of the two references. More specifically, the timing of admixture for the one-reference-weighted LD curve was always significantly lower with ZMA or MAY as the source proxy ($t_{a}=17.48\pm 2.48$ and $t_{a}=18.26\pm 2.36$, respectively) than any other EUT population or ZEB population. Although difficult to prove formally, this may indicate that the TAF founders had heterogeneous EUT ancestry, i.e. one or some of the few founders may have had substantially more Indian Ocean Zebu ancestry than the others.

#### The TAF Recent Size Population History

Figure 2b plots the recent evolution of the effective population size of the TAF population as inferred using GONE (Santiago et al. 2020) with the W50K data set after separating the 1992 (n=12) and 2006 (n=6) samples. The two resulting curves were consistent and both showed a clear bottleneck down to $N_{e}=4.80$ (20 generations ago) and $N_{e}=5.38$ (24 generations ago), respectively, and $N_{e}=4.52$ (22 generations ago) when both samples are combined. Such estimates are remarkably consistent with the historical data referring to five founders in 1871. The timing also coincides with the estimated admixture time (22 generations ago), suggesting that admixture occurred almost simultaneously with the founding of the TAF population, consistent with the above hypothesis from the ALDER results about the possible genetic heterogeneity of the founders. Finally, Fig. 2b shows that the bottleneck was followed by a very steep increase in TAF population size toward the final effective size of about 1,500 individuals, although no evidence of a decline corresponding to the paratuberculosis outbreak reported in the 1950s could be found. It should also be noted, that the above analysis of mitochondrial sequences based on the WGS data we analyzed below is consistent with a very small number of introduced founders, since only one haplogroup (and only two polymorphic positions) was found among the eight individuals.

### Characterization of the Genetic Diversity with WGS Data

We were fortunately able to recover enough high-quality DNA of eight TAF females sampled in 1992 to perform their whole-genome sequencing together with eight ZMA and eight MAY individuals as representative of their Indian Ocean Zebu ancestry (supplementary table S4, Supplementary Material online). We analyzed these 24 newly generated WGS data with publicly available ones for eight JER and eight HOL representing the EUT breeds, choosing data with similar coverage characteristics. Note that the eight WGS TAF females were unrelated to second degree (supplementary fig. S1, Supplementary Material online) except for a single pair of second-degree relatives (TAF_5609 and TAF_5619). As for the SNP genotyping data (see above), we repeated all the analyses presented below without TAF_5619 and obtained very similar results.

#### Within-Population Genetic Diversity

Based on these WGS data, we first characterized the impact of the demographic history on inbreeding and diversity levels in the TAF and compared these with those observed in other populations. Importantly, all these populations have experienced different demographic histories since the establishment of the TAF population. In particular, European cattle have been intensively selected and have undergone a continuous decline of their effective population sizes over recent generations (e.g. Gautier et al. 2010), whereas Indian Ocean Zebu populations have experienced more ancient and less intense bottlenecks (Magnier et al. 2022).

**Fig. 3.**
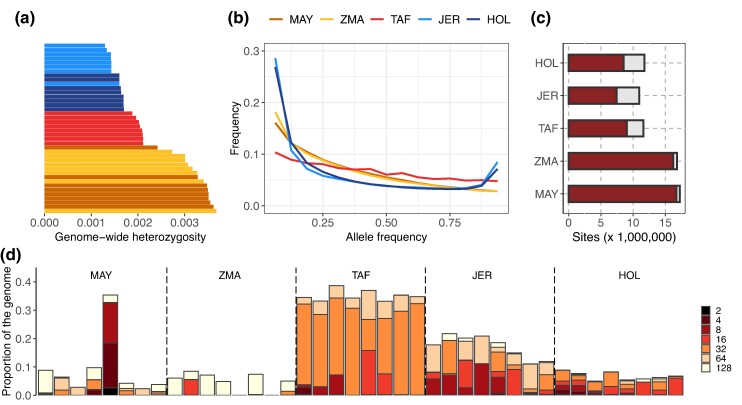
Genetic diversity estimated with WGS data in the TAF populations and populations representative of its European taurine (JER and HOL) and Indian Ocean Zebus (ZMA and MAY) ancestry. a) Genome-wide nucleotide diversity (heterozygosity) of the 40 individuals ranked in increasing order and colored according to their population of origin. b) Site frequency spectrum estimated for the five breeds. c) Number of sites per population that are polymorphic (solid bars) and fixed (open bars) for the derived allele. d) Inbreeding levels, and partitioning of inbreeding in different HBD classes in the different populations. The color code indicates the rate $R_{c}$ of the HBD classes (i.e. the expected length of the HBD segments is equal to $1/R_{c}$ Morgans, with smaller rates and darker colors corresponding to longer and more recent HBD segments).

Despite a very strong founder event, TAF individuals exhibited slightly higher genome-wide heterozygosity than the European breeds JER and HOL, but, as expected, significantly lower than the Indian Ocean Zebus ZMA and MAY (Fig. 3a). Similar conclusions could be drawn when comparing the number of segregating sites, which was almost halved in TAF compared to MAY and ZMA, but (slightly) higher than in HOL or JER (Fig. 3c and supplementary table S7, Supplementary Material online). Similarly, the ranking in terms of fixation levels was reversed due to increased drift in small populations, with four times more fixed variants in TAF compared to MAY or ZMA Zebus, but more than 20% less compared to HOL and JER (supplementary table S8, Supplementary Material online). A striking difference in the TAF was the unfolded site frequency spectrum (SFS), which was flattened compared to other populations (Fig. 3b), with fewer rare alleles and more alleles segregating at higher frequencies. To further characterize the effect of the recent TAF history on the individual levels of inbreeding, we partitioned individual genomes into different classes of autozygous (*aka* homozygous-by-descent or HBD) segments (Druet and Gautier 2017). According to our model, the length of HBD segments is assumed to be exponentially distributed with a class-specific rate (i.e. the expected length is specific to each HBD class). Each HBD class thus corresponds to a distinct group of past ancestors (e.g. long segments correspond to a group of recent ancestors). We observed high levels of inbreeding in the TAF population, averaging 33% (Fig. 3d). Interestingly, autozygosity was concentrated in the HBD class associated with ancestors present about 15 generations in the past (i.e. the HBD class with rate $R_{c}$ equal to 32 and an expected length of about 3 cM). This is fully consistent with the founding event and the demographic history estimated under the GONE model (Santiago et al. 2020), especially if we consider that neighboring classes corresponding to eight ($R_{c}=16$) and 32 ($R_{c}=64$) generations, respectively, are more distant from the founder event. These values were higher than recent levels of inbreeding ($R_{c}\leq 128$) observed in Indian Ocean Zebu (MAY and ZMA) or EUT (HOL and JER) populations, despite intensive selection and reduced effective population size. Strikingly, however, the TAF individuals had few long HBD segments associated with recent ancestors (recent HBD classes had low contributions). This indicates that the population expanded rapidly (the period of reduced $N_{e}$ was short), consistent with demographic estimates, and that most of the inbreeding occurred a few generations after the bottleneck. In contrast, individuals from the two EUT dairy breeds HOL and JER showed higher levels of recent inbreeding, despite management efforts to avoid it. Finally, we compared the partitioning estimated with the 50K SNP genotyping data between the 12 individuals sampled in 1992 and the six individuals sampled in 2006, where the SNP density was sufficient to obtain a good resolution for recent times, up to about class $R_{c}=128$ (representative of ancestors living 64 generations ago), i.e. before the establishment of the TAF population (Druet and Gautier 2017). As shown in supplementary fig. S7, Supplementary Material online, autozygosity levels were stable (from 32.6% to 32.9% on average), but concentrated in more ancient HBD classes for the most recent group of individuals (inbreeding levels in the most recent classes $R_{c}\leq 16$, dropped from 5.9% to 2.1% on average), indicating that ancestors contributing to inbreeding are becoming more distant with time. This confirms that new inbreeding is not generated and that autozygosity is mainly related to the founder event.

#### Genetic Load

**Fig. 4.**
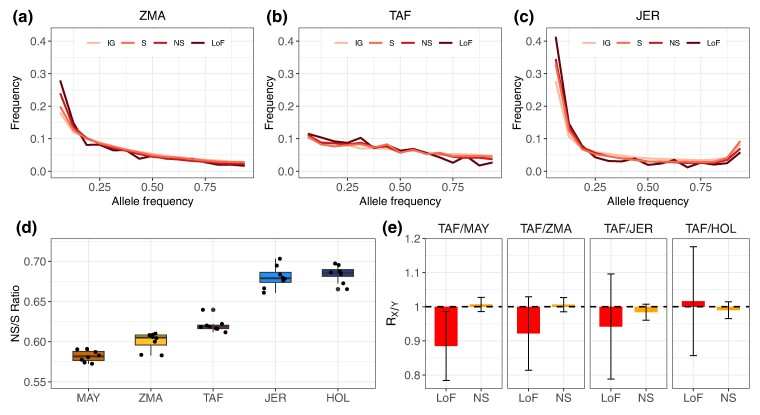
Genetic load in cattle populations. a) to c) Comparison of the SFS for intergenic (IG), synonymous (S), nonsynonymous (NS), and loss-of-function (LoF) variants in the three breeds. d) Ratio of heterozygous NS versus S genotypes per individual. e) $R_{X/Y}$ statistics for comparisons between TAF and other breeds for NS and LoF variants.

Next, we examined the effect of the founder event on the distribution of deleterious variants to investigate whether purifying selection was relaxed or whether some purging occurred as in other species that experienced a severe bottleneck, such as mountain gorillas (Xue et al. 2015) or Alpine ibex (Grossen et al. 2020). To this end, we first compared the proportions of variants in different functional categories, as in Grossen et al. (2020). The TAF was found to have about 5% higher proportions of polymorphic NS (nonsynonymous) variants than MAY and ZMA, but about 10% lower than European cattle (supplementary table S7, Supplementary Material online). The higher proportion of NS variants compared to the two Indian Ocean Zebus suggests that there has been some relaxation of purifying selection, although the proportions of the most deleterious class of variants (i.e. deleterious NS and loss-of-function or LoF) among the segregating sites were always lower (from 1% to 28%) in TAF than in the other four populations. To investigate this further, we compared the SFS for the different classes of variants (Fig. 4a to c). In TAF, the curves were flattened for all categories, whereas in the other populations, the SFS calculated for deleterious variants were enriched in the low frequency classes (supplementary fig. S8, Supplementary Material online). Consequently, there are proportionally fewer NS or LoF variants with frequency >0.25 in the control populations (JER, HOL, MAY, and ZMA), whereas in TAF the proportion of NS and LoF functions is close to that observed for other variant classes (including intergenic variants) up to allele frequencies close to 75% where it starts to decrease. This suggests that NS and LoF variants can reach higher frequencies more often in TAF.

The ratio of the number of heterozygous NS to S genotypes per individual has previously been used as evidence for increased mutation load, for example as a result of domestication (Cruz et al. 2008; Renaut and Rieseberg 2015). We observed that this ratio was only slightly higher in TAF than in MAY and ZMA (Fig. 4d) but remained significantly lower than in JER and HOL, the two intensively selected dairy cattle breeds, suggesting some relaxation of selective constraints in TAF. Next, following Smeds and Ellegren (2023), we divided the genetic load into masked and realized load, estimated as the number of heterozygous and fixed derived variants per individual, respectively. The TAF population showed a slightly higher masked load than HOL and JER for all classes of variants, but it remained well below the levels observed in MAY and ZMA (supplementary fig. S9, Supplementary Material online). The trends were reversed for the realized load, which may be the result of the high inbreeding levels and fixation rate observed in TAF (and HOL and JER) compared to MAY and ZMA. In general, the number of homozygous genotypes (realized load) is more relevant when deleterious effects are recessive, whereas the number of derived alleles per genome would be more relevant for additive effects and is predicted to be insensitive to demographic history for neutral alleles (Mooney et al. 2023). Interestingly, we observed that the frequencies of derived intergenic variants in our populations were all very similar, ranging from 0.277 to 0.279 (with three populations at 0.278), consistent with expectations for neutral alleles. This suggests that our data processing, including variant filtering and polarization, was implemented reliably (Mooney et al. 2023). We, therefore, confidently used the AFs to compute the $R_{X/Y}$ statistic (Do et al. 2015), a relative measure that indicates whether deleterious variants in population *X* are under relaxed ($R_{X/Y}>1$) or stronger ($R_{X/Y}<1$) purifying selection with respect to population *Y*. As shown in Fig. 4e and in agreement with the proportions of NS variants in the different populations (supplementary table S7, Supplementary Material online) and other statistics described above, $R_{\mathrm{TAF}/Y}$ computed with NS was slightly >1 for comparisons with Indian Ocean Zebus (Y=MAY and Y=ZMA) and <1 for comparisons with EUT populations (Y=HOL and Y=JER), although the value of 1 could not be excluded from the block-jackknife 95% CI in all comparisons. For LoF variants (Fig. 4e), $R_{\mathrm{TAF}/Y}$ were <1 for all comparisons except for Y=HOL (but only significant for Y=MAY). This suggests a slightly higher level of purging for this category of variants in TAF.

### Adaptive History of the TAF Population

#### Genetic Offset

**Fig. 5.**
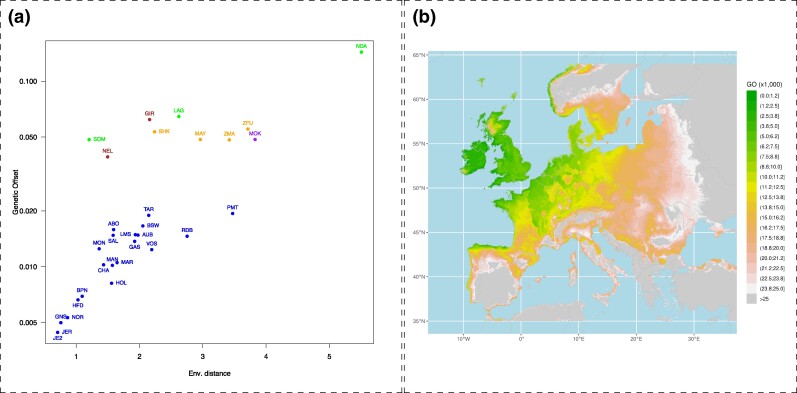
Estimation of domestic bovine maladaptation to the Amsterdam island environment using GO statistics. a) Estimated GO between birthplace environments of 31 populations from the W50K data set as a function of environmental distance. b) Estimated GO across Europe.

In order to assess the potential environmental challenges that the bioclimatic condition of Amsterdam Island may represent for different (adapted) cattle populations originating from different regions, i.e. their relative maladaptation or preadaptation, we used the genetic offset (GO) statistic (Capblancq et al. 2020). For this purpose, we relied on the BayPass Gene–Environment Association (GEA) model (Gautier 2015) to summarize the relationship between seven bioclimatic PCs (resulting from a PCA of 19 bioclimatic covariates) and the genomic composition of the 32 populations represented in the W50K data set. We then estimated the GO between the Amsterdam island environment and the birthplace environment of each of the 31 (non-TAF) populations (Fig. 5a), following Gain et al. (2023). Interestingly, the lowest GO was obtained for the environment associated with the birthplace of JE2 (and JER), which was also the one with the smallest environmental distance, i.e. calculated without considering the association with the bovine (adaptive) genomic composition (Fig. 5). The geographically close environment of the island of Guernsey and Normandy that were associated with the birthplaces GNS and NOR also led to small GO, only 12.8% and 20.1% higher than JE2 and JER, respectively. Figure 5b further describes the distribution of GO over European environments with respect to the Amsterdam island. Within this broader range, the island of Jersey was also found to be among the regions with the lowest GO, along with the coastal regions of Brittany, Great Britain, Ireland, and northern Spain. As expected, GO was highest for environments associated with Zebu or African cattle originating from tropical or subtropical regions (Fig. 5a) but also for southern and continental European environments (Fig. 5b). Overall, this suggests that the climatic conditions on the island of Jersey (or closely related regions) and those on the island of Amsterdam were not so different, and the populations that originated from this region, and that according to demographic inference contributed the most to the ancestry of the TAF population, may have been largely preadapted.

#### Genomic Prediction of Adult Height Reveals No Evidence for Dwarfism in TAF

**Fig. 6.**
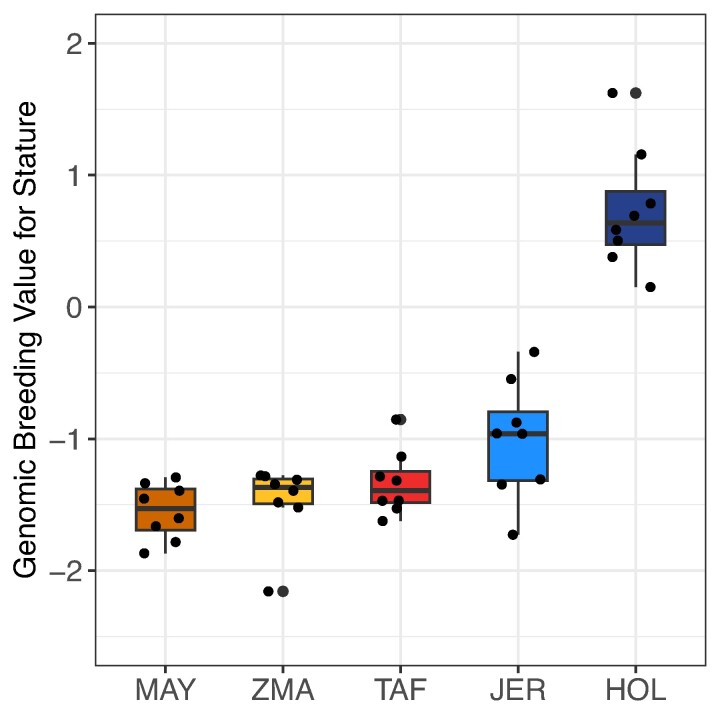
Distribution of genomic prediction of individual height breeding values for each population based on the estimated effects of 105 known variants affecting stature in cattle (Bouwman et al. 2018) genotyped with our analyzed WGS data (n=8 individuals per population).

Although adapting to the new environment did not appear to be a major challenge, we looked for evidence of selection in the TAF population. For example, based on scarce phenotyping data, Rozzi and Lomolino (2017) argued for rapid dwarfism in this population. If true, this process would have taken place in only a few generations and might have left some strong genomic signatures of selection, at least for the major genetic variants controlling bovine height which would have been polymorphic among the founders. However, our demographic inference showed that the TAF founders are closely related to two populations with short stature (Madagascar Zebu and Jersey individuals). Thus, they may not have been dwarfed as suggested, since the dimensions reported in Rozzi and Lomolino (2017) for TAF cattle are actually consistent with those of the Zebu and Jersey populations, and the TAF would only be short compared to larger cattle breeds (e.g. Holstein). To confirm this, we investigated whether known alleles contributing to short stature were enriched in the population using 164 variants from a recent cattle meta-analysis (Bouwman et al. 2018), and we used their estimated genetic effects to predict the height of the sequenced individuals. This amounts to weight each allele by its predicted effect on stature. We were able to find 105 of these variants in our sequenced individuals (eight per population). As shown in Fig. 6, the mean breeding value for (standardized) height was equal to −1.34 for the TAF individuals (ranging from −1.62 to −0.85), and was found to be intermediate between those observed in ZMA (mean of −1.47 with values ranging from −2.16 to −1.28) and in JER (mean of −1.01 with values ranging from −1.73 to −0.34), and substantially lower than for the HOL breed. Accordingly, we observed that the short allele of the PLAG1 mutation (Karim et al. 2011) was fixed in all TAF but also in all JER individuals. Thus, there is no evidence that the small size of the TAF individuals is due to (polygenic) selection for “short” alleles.

#### Detecting Footprints of Selection on a Dense Haplotype Data Set

Finally, to provide a global genomic description of the adaptive response of the TAF population in its newly colonized environment, we searched for footprints of positive selection using extended haplotype homozygosity (EHH) based tests, considering either the iHS (Voight et al. 2006) or the Rsb (Tang et al. 2007) statistics for within-population and pairwise comparisons, respectively. The iHS and Rsb based tests are complementary and serve different purposes, the iHS being better suitable for detecting recent and strong selective sweeps where the underlying variants are still segregating in the population, while the Rsb is more focused on detecting localized and potentially older (but still strong) selective events that lead to fixation of the causal variant in one or the other population. For Rsb, we focused here on TAF-specific signals by searching for extended site-specific homozygosity in the TAF with respect to either JER (${\text{Rsb}}_{\mathrm{TAF}/JER}$) or ZMA (${\text{Rsb}}_{\mathrm{TAF}/ZMA}$), considered as source population proxies, using unilateral tests of extremely positive values. To improve mapping resolution, we analyzed a data set combining high-density (>770,000 SNPs) SNP genotyping data from the Illumina BovineHD assay for 23 ZMA and 30 JER individuals (Sempéré et al. 2015; Magnier et al. 2022) with the subset of 50K SNP data (most of which are shared with the BovineHD assay) for the 18 TAF individuals. We then imputed (or directly genotyped for the eight sequenced TAF individuals) the missing BovineHD genotypes in the TAF using the available WGS data (see M&M section). After filtering and phasing, the analyzed data set consisted of 142 haplotypes (36 TAF, 46 ZMA, and 60 JER) per autosome, totaling 530,769 SNPs (ranging from 8,842 to 33,710 for chromosomes 25 and 1, respectively). In total we could identify 21 significant regions, 12 (ranging from 76.90 to 1,355 kb in length) associated with the within TAF iHS, and two and eight with the ${\text{Rsb}}_{\mathrm{TAF}/JER}$ (83.10 and 297.7 kb in length) and ${\text{Rsb}}_{\mathrm{TAF}/ZMA}$ (ranging from 196.7 to 1,352 kb), respectively (Table 1). Note that two regions, identified with iHS and ${\text{Rsb}}_{\mathrm{TAF}/JER}$, overlapped and led to the identification of the same candidate genes (based on the distance to the peak statistic). More signals were identified with ${\text{Rsb}}_{\mathrm{TAF}/ZMA}$ than ${\text{Rsb}}_{\mathrm{TAF}/JER}$, which may be related to the lower recent historical $N_{e}$ in JER compared to ZMA, resulting in higher EHH thereby limiting the detection power. In addition, we noticed that two ${\text{Rsb}}_{\mathrm{TAF}/ZMA}$ regions overlapped with those detected based on ${\text{Rsb}}_{\mathrm{TAF}/ZMA}$ statistics (supplementary table S9 and fig. S10, Supplementary Material online). For one of these regions, the identified candidates (and corresponding statistic peaks) were more than 1 Mb apart. We, therefore, considered them to be separate signals. However, for the other region, the two windows pinpointed the same candidate gene (SLC4A4), suggesting that this region had already been under selection before the establishment of the TAF population (in the ancestral JER-related population) and this nonspecific TAF signal was further disregarded.

**Table 1:** Description of the regions containing footprints of selection based on the iHS, ${\text{Rsb}}_{\mathrm{TAF}/JER}$and ${\text{Rsb}}_{\mathrm{TAF}/ZMA}$ EHH-based tests

ID	Test	Win. pos.	Win. size in kb	Peak pos. in kb	Nearest gene
		(chr:start to end in kb)	(Nsnp)	(Stat. value)	(Pos. in kb ; dist. from peak)
1	${\text{iHS}}_{TAF}$	3:43,306 to 43,463	156.7 (19)	43,343 (3.87)	AGL (43,352 to 43,353 ; 8)
2	${\text{iHS}}_{TAF}$	9:79,703 to 80,097	394.0 (35)	79,867 (4.77)	ADGRG6 (79,770 to 79,923 ; 0)
3	${\text{iHS}}_{TAF}$	9:81,292 to 81,834	542.4 (62)	81,637 (3.20)	UTRN (81,652 to 82,200 ; 15)
4	${\text{iHS}}_{TAF}$	10:67,993 to 68,586	593.6 (33)	68,209 (3.24)	LOC112448598 (68,272 to 68,272 ; 62)
5	${\text{iHS}}_{TAF}$	10:73,678 to 74,139	461.3 (56)	73,955 (3.08)	SYT16 (73,937 to 74,244 ; 0)
6	${\text{iHS}}_{TAF}$	10:76,116 to 76,609	492.9 (22)	76,145 (2.94)	SYNE2 (76,063 to 76,387 ; 0)
7	${\text{iHS}}_{TAF}$	15:34,973 to 35,050	76.90 (16)	34,982 (3.33)	USH1C (34,963 to 35,012 ; 0)
8	${\text{iHS}}_{TAF}$	16:58,363 to 59,261	897.7 (38)	58,537 (6.27)	BRINP2 (58,417 to 58,560 ; 0)
9	${\text{iHS}}_{TAF}$	23:23,433 to 23,866	432.5 (36)	23,647 (3.51)	TRNAY to AUA (23,515 to 23,515 ; 132)
	${\text{Rsb}}_{TAF/JER}$	23:23,314 to 23,611	297.7 (76)	23,555 (3.27)	TRNAY-AUA (23,515 to 23,515 ; 40)
10	${\text{iHS}}_{TAF}$	23:46,236 to 46,627	390.5 (25)	46,378 (5.15)	OFCC1 (45,984 to 46,226 ; 151)
11	${\text{iHS}}_{TAF}$	23:47,230 to 47,618	387.5 (23)	47,400 (3.61)	BLOC1S5 (47,393 to 47,417 ; 0)
12	${\text{iHS}}_{TAF}$	29:27,218 to 28,574	1,355 (28)	27,819 (2.75)	OR8B8 (27,808 to 27,809 ; 10)
13	${\text{Rsb}}_{TAF/JER}$	14:49,264 to 49,347	83.10 (22)	49,298 (2.91)	TRPS1 (48,630 to 48,909 ; 389)
*14^⋆^*	${\text{Rsb}}_{TAF/ZMA}$	*6:86,541 to 87,032*	*491.5 (125)*	*86,744 (4.19)*	*SLC4A4 (86,449 to 86,813 ; 0)*
15	${\text{Rsb}}_{TAF/ZMA}$	6:101,018 to 101,215	196.7 (54)	101,032 (4.96)	MAPK10 (100,908 to 101,530 ; 0)
16	${\text{Rsb}}_{TAF/ZMA}$	7:62,509 to 62,917	407.8 (133)	62,534 (3.47)	SLC36A3 (62,521 to 62,545 ; 0)
17	${\text{Rsb}}_{TAF/ZMA}$	8:88,183 to 88,415	232.8 (66)	88,220 (4.34)	LOC132345997 (88,227 to 88,231 ; 7)
*18^⋆^*	${\text{Rsb}}_{TAF/ZMA}$	*20:24,573 to 24,888*	*314.7 (75)*	*24,690 (3.83)*	*HSPB3 (24,638 to 24,639 ; 51)*
19	${\text{Rsb}}_{TAF/ZMA}$	21:35,024 to 36,449	1,425 (465)	35,244 (4.76)	STXBP6 (35,025 to 35,306 ; 0)
20	${\text{Rsb}}_{TAF/ZMA}$	23:23,810 to 24,896	1,086 (360)	23,873 (4.29)	PKHD1 (24,071 to 24,513 ; 198)
21	${\text{Rsb}}_{TAF/ZMA}$	23:25,197 to 26,549	1,352 (198)	25,333 (4.10)	GCM1 (25,311 to 25,333 ; 0)

The two nonspecific TAF regions (#14 and #18) that overlap with those identified using ${\text{Rsb}}_{\mathrm{JER}/\mathrm{ZMA}}$ (supplementary table S9, Supplementary Material online) are in italics and highlighted with a ^⋆^.

The remaining 20 candidate genes were then subjected to functional annotation using the Ingenuity Pathway Analysis (IPA) software (Ingenuity Systems, Inc. 2023). Three genes (i.e. LOC112448598, TRNAY-AUA, and LOC132345997) that were not recognized in the IPA database (IPKB) were removed from the analysis. The top five diseases and biological functions significantly enriched by the analysis of the 17 annotated candidate genes (supplementary table S10, Supplementary Material online) were related to the nervous system (i.e. Neurological Disease and Nervous System Development and Function), and to tissue, organ and organism development (i.e. Organismal Injury and Abnormalities, Cell Morphology, and Tissue Morphology). More specifically, 15 of the 17 genes (i.e. ADGRG6, AGL, BLOC1S5, GCM1, HSPB3, MAPK10, SYNE2, USH1C, UTRN, OR8B8, PKHD1, SLC36A3, SYNE2, SYT16, TRPS1) are involved in nervous system function and/or its development. Accordingly, among the top five canonical pathways identified (supplementary table S11, Supplementary Material online), four were also found to be related to the nervous system either directly (for “EGR2 and SOX10-mediated initiation of Schwann cell myelination,” Schwann cells are derived from the neural crest; “Agrin Interactions at Neuromuscular Junction” and “SNARE Signaling Pathway”) or indirectly (for “Interleukin-6 signaling,” IL6 being a major cytokine in the central nervous system).

## Discussion

The central goal of this study was to gain a deeper understanding of the process of establishing a large domestic mammal population from a small number of founders by studying the particular case of the TAF feral cattle population that lived on Amsterdam Island. To this end, we inferred its demographic history and characterized its genetic makeup up to its complete and questionable eradication in 2010. Despite challenges, we were able to retrieve DNA samples for 18 individuals collected in 1992 and 2006, which we could genotype with a medium density commercial SNP assay, and for eight of them whole-genome sequenced. The sample had some limitations, and in particular, we were unfortunately unable to obtain genomic information for the Y chromosome, which could have helped gain insights into the paternal lineage. However, our newly generated data, thanks to recent methodological advances in population genomics inference methods, allowed us to uncover intriguing details about the origins and history of the population and to formulate plausible hypotheses explaining the success of the population establishment.

The estimation of the recent historical effective population size of the TAF population from genetic data supports a strong founder effect, down to five individuals, 22 generations before sampling, which corresponds to the late 19th century. These results confirm the accuracy of historical records about the foundation of the population by a few individuals brought to the island in 1871 and contradict other alternative hypotheses about the alleged contribution of cattle introduced by seafarers during the 18th and early 19th centuries (Lesel 1969; Micol and Jouventin 1995). Interestingly, using 1992 as the mean birth year of the analyzed individuals (supplementary table S1, Supplementary Material online) and 1871 as the founding date, such a dating can provide an indirect estimate of the cattle generation time of 5.5 years in free living conditions, which is consistent with the value classically accepted in the literature, as we discussed in Magnier et al. (2022).

The construction of admixture graph (Patterson et al. 2012; Gautier et al. 2022) indicated that two main ancestries contributed to the TAF population, consisting of about 75% to European taurine cattle, related to the present-day Jersey breed, and for the remaining to Indian Ocean Zebu, related to the present-day Zebu of the islands of Madagascar and Mayotte. The timing of the corresponding admixture event was contemporaneous with the founding bottleneck, and detailed examination suggested the founders themselves may have been of heterogeneous ancestry. Such a result is consistent with the fact that Heurtin, the farmer who attempted to settle in the Amsterdam island, chose his animals from among those present on La Réunion island, which were related to European cattle breeds (e.g. Jersey, “Pie-Noir bretonne” from Brittany, and “Grise des Alpes” and Tarine both from the French Alps) and to Indian Ocean Zebus (Lesel 1969). It is further tempting to speculate that he favored individuals of Jersey or Brittany breed type, as they were best suited to survive in the harsh conditions of the Amsterdam island. The selected individuals may have had varying degrees of Indian Ocean Zebu ancestry due to more or less distant Zebu ancestors, as a result of mixing on La Réunion island. Overall, the demographic inference of the genetic history of the TAF population confirmed that this population represents a rare example of a successfully established large mammal from a recent (22 generations), brief (only a few generations), and extreme (only five founders) population bottleneck that has rapidly thrived in a seemingly challenging environment.

We further performed a detailed comparative analysis of genetic diversity at the whole-genome level of the TAF population based on the WGS data of eight individuals. Despite high levels of individual inbreeding (∼30%), the TAF population showed no evidence of strong genetic diversity reduction, these two attributes being usually inversely related. The levels of inbreeding observed in TAF are within the range (15% to 45%) reported in endangered or recently recovered populations of various mammalian species (Xue et al. 2015; Abascal et al. 2016; Robinson et al. 2016, 2019; Druet et al. 2020; Grossen et al. 2020; Kyriazis et al. 2023; Mooney et al. 2023). However, the distribution of HBD segments is different, with a high proportion of relatively short HBD segments (tracing back to the founding event) and no evidence of more recent inbreeding that would have generated longer HBD segments. This is consistent with a rapid expansion scenario, inbreeding levels related to the extremely small number of founders being stabilized shortly after the bottleneck. Combined with the admixed origin of the founders, the demographic history of the TAF population may thus explain its relatively high overall heterozygosity ($≃2\times 10^{-3}$) compared to that ($<5\times 10^{-4}$) reported in most of the aforementioned endangered populations with similar levels of inbreeding. In fact, loss of genetic diversity has been most commonly observed in populations that have experienced small effective population sizes over many generations, while bottlenecks of short duration have been shown to have minimal effects on genetic diversity (Robinson et al. 2023). The resulting preservation of the initial genetic diversity present in the founders, even in small numbers, is thus consistent with one of the solutions to the paradox of invasion regarding the actually limited impoverishment of genetic diversity (Estoup et al. 2016).

Population bottlenecks leave other genomic footprints such as high fixation levels (e.g. Smeds and Ellegren 2023) and a flattening of the SFS (e.g. Alves et al. 2022), which we also clearly observed in the TAF population. Remarkably, we also found that the SFS was flattened for all classes of variants, including deleterious NS and LoF that both exhibited a lower proportion of singletons (relative to intergenic variants) than in the other European taurine or Indian Ocean Zebu populations. This suggests that purifying selection against deleterious variants may have been relaxed in the TAF cattle, resulting in some accumulation of the genetic load, as previously observed in domestic species (Cruz et al. 2008; Marsden et al. 2016; Bosse et al. 2019) and in several of the wild populations mentioned above. Accordingly, the proportions of segregating NS variants (a proxy for mildly deleterious variants) and the NS/S ratio were found to be higher in the TAF population compared to the two Zebu populations, and the corresponding $R_{X/Y}$ statistic also pointed to a slight excess of NS variants. It should be noted that all these statistics were less extreme in the TAF population than in the European breeds JER and HOL, likely because these dairy cattle have been subjected to intense selection for several decades and have maintained an effective population size of only a few hundreds of individuals over the last tens of generations (e.g. Gautier et al. 2010).

Despite indications of a relaxation of purifying selection observed at the genomic level, observers reported individuals in excellent health and condition, at least in the highest part of the island (Lesel 1969). Furthermore, the population was able to expand even after the sharp decline observed in 1953 following an infectious disease outbreak, or to maintain itself after regulation culling in 1988 and 1992 (see Fig. 2 legend). Purging of highly deleterious variants is sometimes invoked to explain the absence of fitness loss due to inbreeding depression in small populations (Bouzat 2010). When focusing on LoF (a proxy for highly deleterious) variants, although the lower proportion of segregating variants (and a $R_{X/Y}$ slightly <1) may hint at mild purging, the higher proportions of fixation and the flattened SFS observed in the TAF population rather suggest a relaxation of purifying selection. In other words, we found no clear evidence of purging of the most deleterious variants in this population, which is actually consistent with the short duration of its bottleneck. Indeed, longer periods of reduced population size and slower rates of inbreeding levels are required for purging to be effective (e.g. Bouzat 2010; Balick et al. 2015; Robinson et al. 2023). In summary, the observed effects of the extreme bottleneck experienced by the TAF population on genetic diversity and genetic load align with its age, its strength (e.g. high inbreeding and fixation levels and relaxation of purifying selection) and its short duration (small effect on heterozygosity levels and no clear evidence of purging).

If the extreme bottleneck did not pose an insurmountable demographic challenge, the TAF population still had to face and adapt to extreme environmental conditions. Indeed, the few founding individuals may have benefited from the farmer’s care for only a few months before being left alone. The remote location of the subantarctic island of Amsterdam in the Southern Ocean exposes it to harsh and unpredictable weather patterns. The island is frequently buffeted by strong winds, sometimes reaching hurricane force, and the persistent cold (with temperatures often hovering around freezing) adds to the challenging conditions. In addition, the island’s isolation and limited resources, particularly fresh water, add to the harshness of the environment. Nevertheless, the estimation of domestic bovine maladaptation in the form of GO (Capblancq et al. 2020), calculated between the climatic variables of the Amsterdam island and those of the other breeds’ locations showed that the GO for the environmental conditions associated with the geographical origin (i.e. Channel Islands and North of Brittany) of the main European taurine ancestry of the TAF population was among the lowest (i.e. less challenging). Although the absolute value of GO is difficult to interpret per se, it has been shown to be directly related to average population fitness (Gain et al. 2023) and to the establishment probability of invasive populations (Camus et al. 2024). Hence, the GO analyses suggest that the adaptive challenge on Amsterdam island was limited given the inferred origin of the TAF population. In other words, the successful establishment of the TAF population can potentially be explained by a form of preadaptation of its founders to the local climatic conditions due to their predominant European taurine ancestry. The TAF population thus provides a good example of the preadaptation of a nonindigenous population, able to survive and reproduce in local environmental conditions close to those of its place of origin. This is consistent with the so-called matching hypothesis, despite an extremely low propagule pressure (Sol 2007).

Recent studies have highlighted the “island syndrome,” which includes the frequent dwarfism of large-bodied mammals on islands (Rozzi et al. 2023). Since the TAF is a rather short cattle population, it was tempting to speculate that its short stature resulted from a rapid dwarfism (Rozzi and Lomolino 2017) and that the feralization process and food restriction on the island may indeed have favored selection for small animals. However, our results argue against such an insular dwarfism syndrome in the TAF population. First, the demographic inference strongly suggests that the small size of TAF individuals, with a wither height of 134 and 113 cm (Lesel 1969) and an average weight of 389.6kg±42.8 and 293kg±45.8 (Berteaux and Micol 1992) for males and females, could more parsimoniously be directly related to the small format of the populations from which they originate. The JER breed is among the smallest of all dairy breeds with an average female wither height of around 120 cm and female weight of 375 kg. In addition, old but detailed reports confirm that the most numerous cattle population that lived in Brittany (to which the current BPN is related) at the end of the 19th was notoriously small, with a reported average size of 100 to 110 cm (de Lapparent 1902). Likewise, Indian Ocean Zebus are of small format with, for example, an average male wither height of 110 cm and weight of 240 kg measured in the ZMA (Zafindrajaona and Lauvergne 1993). Second, at the genome level, our estimates of breeding values for stature based on estimated effects for 105 variants obtained in an extended meta-analysis that included several cattle breeds (Bouwman et al. 2018) did not provide evidence for strong (polygenic) selection for short stature. Finally, the observed small size of TAF cattle may also be partly due to some degree of inbreeding depression, which has been reported for stature in cattle and other mammals (e.g. Brzeski et al. 2014; Yengo et al. 2017; Naji et al. 2024).

Although the environmental conditions may not have been as challenging, the living conditions of the TAF population changed dramatically and rapidly. This led to a complete feralization of the initially domesticated animals in just a few generations. To complete the genomic characterization of the population, we, therefore, examined the genomic response of the TAF population to the new adaptive constraints it encountered. Strikingly, the majority of candidate genes we were able to identify within the footprints of selection were annotated to be involved in nervous system function and development. We can view these results as consistent with the brain size–environmental change hypothesis, which states that large brains in mammals are associated with an enhanced behavioral flexibility, which may confer advantages to individuals by improving their fitness in the face of novel environmental conditions (Sol et al. 2008). They are also consistent with the behavioral modification of TAF individuals that may have accompanied and contributed to the rise of the population on the island and its feralization. Several observers have noted a clear and complex social organization of the TAF, similar to that of wild bovids, with matrilineally structured groups consisting mostly of females and young to subadult males; geographically separated groups consisting exclusively of adult and/or subadult males; and mixed groups usually formed at the beginning of the reproductive season by incorporating adult males into the groups of females (Daycard 1990). Such a complex social structuring, that accompanied feralization, is also supported by the estimated negative population $F_{\mathrm{IS}}$ (Chikhi and Parreira 2015) and the close relatedness of some samples, although our sample size remained limited. Observers also reported that the TAF individuals had become fierce and phenotypically, there was a clear and impressive diversification of color patterns in all the individuals (Lesel 1969). If feralization cannot be viewed as a mere reversal of domestication (Gering et al. 2019), these observations, and our results showing the apparent importance of nervous system function in TAF adaptation reveal common features between the feralization process in the TAF population (fierceness) and the domestication syndrome studied in domesticated mammals (tameness), mobilizing some genes involved in the neural crest development (Wilkins et al. 2014). They also highlight the likely polygenic nature of complex traits involved in feralization, and the role of standing variation in the rapid adaptation of the TAF population to the wild. Indeed, this may explain how the variants underlying a feralization that lasted only a few generations were still segregating in the domestic founders.

The TAF population represents a remarkable resource of a domestic population that was able to colonize and thrive in a challenging environment, recovering from only a handful of individuals. The population quickly became feral, developing new abilities rendering it adapted to an harsh environment. Unfortunately, despite a convincing and conclusive management policy to allow cohabitation with endemic species, this population was eradicated in 2010. Moreover, no effort was made to conserve some individuals or even, at the very least, to keep samples indicating that those who promoted this eradication, including biologists, considered it a nuisance without any scientific interest. However, we hope that this article will convince the reader of the opposite and contribute to a more careful reflection before eradicating feral populations. The data and analyses we present will help to preserve a trace and a legacy of the incomparable resource that this population represented for the scientific community.

## Materials and Methods

### Genetic Data

#### New Sample Origin, Genotyping, and Sequencing Data

Blood samples for 18 different bovine individuals from the Amsterdam island (TAF) were collected during two sampling campaigns in 1992 (*n*=12 females) and 2006 (n=6, three females and three males) and frozen at −20 ^∘^C before being transferred to the former LGbC laboratory (INRA, Jouy-en-Josas) and to the LaboGENA genotyping platform (Jouy-en-Josas, France), respectively (supplementary table S1, Supplementary Material online). Genomic DNA for the 12 females collected in 1992 were extracted in March 1994 using the protocol described in Jeanpierre (1987) which allowed long-time preservation of DNA integrity at −20 ^∘^C. The genomic DNA of the six individuals collected in 2006 was extracted at the LaboGENA platform for genotyping purposes and was unfortunately neither stored nor returned after the test. All the 18 TAF individuals were genotyped on the Illumina BovineSNP50 chip assay v2 (Matukumalli et al. 2009) at the LaboGENA platform (Jouy-en-Josas, France) using manufacturer recommendations (Illumina 2016), in 2014 for the 12 individuals collected in 1992 and in 2010 for the six individuals collected in 2006. Together with this latter TAF individuals, 31 individuals belonging to the Moka Zebu breed (MOK) sampled in 2010 in La Réunion island were also genotyped on the same assay. All these newly generated genotyping data (n=49 individuals in total) have been made publicly available in the WIDDE repository (Sempéré et al. 2015). Finally, 8 of the 12 TAF females collected in 1992 and for which enough DNA was still available were further paired-end sequenced (2×150 nt) on a HiSeqX Illumina sequencer at the Macrogen commercial platform (Macrogen Inc., Seoul, South Korea). For the purpose of this study, we also sequenced 16 Zebus from Mayotte (n=8) and Madagascar (n=8) islands that were collected in 2017 and 1989, respectively, and that were among the individuals previously described and genotyped in Magnier et al. (2022) and Gautier et al. (2009). Thirteen (eight MAY and five ZMA) were paired-end sequenced on a HiSeq 2500 (n=5 with 2×125 nt) or a NovaSeq 6000 (n=8 with 2×150 nt) Illumina sequencer at the MGX platform (Montpellier, France). The three remaining ZMA individuals were paired-end sequenced (2×150 nt) on a NovaSeq 6000 Illumina sequencer at the Genoscope platform (Evry, France) (supplementary tables S4 and S2, Supplementary Material online). All the newly generated sequencing data for the eight TAF, the eight MAY and the eight ZMA individuals were deposited in the NCBI Sequence Read Archive repository under the BioProject accession number PRJNA1010533 (supplementary table S4, Supplementary Material online).

#### The 50K SNP Genotyping Data Set (W50K) Representative of Worldwide Cattle Genetic Diversity

To explore the genetic relationship of the TAF population with other bovine population and infer its origin, the newly generated data obtained with the BovineSNP50 assay were combined with publicly available data from populations representing the worldwide cattle diversity (Matukumalli et al. 2009; Gautier et al. 2010) and that are stored in the WIDDE database (Sempéré et al. 2015). As detailed in supplementary table S2, Supplementary Material online and represented in Fig. 3a, the resulting W50K data set finally consists of 876 individuals from 32 different populations. Genotyping data were filtered using WIDDE utilities (Sempéré et al. 2015), only retaining individuals with a SNP genotyping call rate >95%. Likewise, we only retained SNPs with an individual genotyping call rate >75% in all population samples (leading to an overall genotyping call rate >90%) and that mapped to the latest ARS-UCD1.2 (aka *bosTau9*) bovine genome assembly (Rosen et al. 2020). In addition, we discarded SNPs with a minor allele frequency (MAF) <0.1% over the entire data set or with a highly significant departure of individual genotype frequencies from Hardy-Weinberg equilibrium expectations ($P<10^{-4}$) in at least one population. The W50K data set finally comprised 40,484 SNPs including 40,426 autosomal SNPs (from 736 on chr. 8 to 2,628 on chr. 1).

#### Whole-Genome Sequencing Data Processing, Variant Calling, and Annotation

To provide a comprehensive genome-wide analysis of TAF genetic diversity and compare it with the most closely related populations as inferred from population genetics analyses on the W50K genotyping data, we analyzed the newly generated WGS data for the eight TAF, eight ZMA, and eight MAY together with eight Jersey and eight Holstein publicly available WGS data (Daetwyler et al. 2014) that displayed similar coverage (supplementary table S4, Supplementary Material online). To infer the ancestral state of the identified variable position, we used WGS data for one American bison (*Bison bison*), one European bison (*Bison bonasus*), one Gaur (*Bos gaurus*), and one Banteng (*Bos javanicus*) available from Wu et al. (2019). Processing of all the WGS data mostly followed the “1000 Bull Genomes” analysis guidelines (version of 2018/06/18) (Daetwyler et al. 2014). Briefly, adaptors were removed from the sequencing reads that were trimmed and filtered using Trimmomatic v0.38 (Bolger et al. 2014) that was run with options LEADING:20 , TRAILING:20, SLIDINGWINDOW:3:15, AVGQUAL:20, MINLEN:35, and ILLUMINACLIP with the TruSeq3-PE.fa:2:30:3:1 adaptor file. The program fastp v0.19.4 (Chen et al. 2018) was further run with default options on the resulting fastq files mainly to provide statistics for Quality Check and also to (marginally) improve adapter removal. After filtering, paired reads were aligned onto the ARS-UCD1.2_Btau5.0.1Y whole-genome assembly that combined the ARS-UCD1.2 (for autosomes and the X chromosome) (Rosen et al. 2020) and the Btau5.0.1 Y chromosome assemblies (Bellott et al. 2014), using the program bwa mem (Li 2013) run with default options. PCR and optical duplicates were subsequently marked using the command MarkDuplicates from Picard 2.18.2 (Broad Institute 2018). Base quality scores were further recalibrated (BQSR step) with the BaseRecalibrator> tool from GATK (v4.2.6.1) (McKenna et al. 2010) using a catalog of 110,270,189 known variants from the 1000 Bull Genome project (Daetwyler et al. 2014) as a recalibration file named ARS1.2PlusY_BQSR.vcf . After BQSR, we performed variant calling using GATK ’s HaplotypeCaller setting the ploidy to one for the mitochondria and for the X chromosome in males. A multisample vcf file was then generated for each chromosome (and the mitochondria) by combining all the 40 resulting gvcf files for bovine individuals with the GATK ’s CombineGVCFs and GenotypeGVCFs tools.

Finally, mitochondrial haplogroups from each individual were inferred using MitoToolPy (Peng et al. 2015) based on the “treeFile” provided by Dorji et al. (2022) for the ARS_UCD1.2 assembly. For each individual, a fasta file was obtained by extracting genotypes from the VCF file using the consensus command from bcftools v1.10.2 and using the reference genome fasta file.

### Population Genetics Structure

#### F-Statistics Computation

The Wright fixation indexes $F_{IT}$, $F_{\mathrm{ST}}$, and $F_{\mathrm{IS}}$ were estimated using a custom implementation of the estimator proposed by Weir and Cockerham (1984) under an analysis of variance framework (see also Weir 1996, p. 176–179) between all pairs or over all the samples. Within-population $F_{\mathrm{IS}}$ were estimated following Weir (1996, p. 80) and heterozygosities were estimated with the compute.fstats function of the R package *poolfstat* (v2.2.0) (Gautier et al. 2022). Standard errors (SE) and 95% CI (as ±1.96 SE) of the different statistics values were estimated using a block-jackknife approach (Busing et al. 1999; Gautier et al. 2022). This here consisted of dividing the genome into contiguous chunks of 250 SNPs, leading to 150 blocks of 15.3 Mb on average (from 10.6 to 21.4 Mb), and then removing each block in turn to quantify the variability of the estimator among the 150 corresponding estimates.

#### Exploratory Analyses

The neighbor-joining trees (Saitou and Nei 1987) were computed using the nj function of R package ape (Paradis et al. 2004) based on the matrix of ASD between all pairs of individuals following (Gautier et al. 2010). PCA of individual SNP genotyping data was carried out as described in Patterson et al. (2006) using the svd function of the R package base (R Core Team 2017). To provide an alternative description of the structuring of genetic diversity, unsupervised genotype-based hierarchical clustering of the individuals was carried out using the maximum-likelihood method implemented in the ADMIXTURE (v1.06) software (Alexander et al. 2009). Results were visualized with custom functions in the R environment (R Core Team 2017).

#### Relationship Inference

The program King (v2.3.2) (Manichaikul et al. 2010) was used to infer relationship among pairs of TAF individuals based on autosomal genotyping data. Inference relied on both the estimates of kinship coefficient and IBD-segment sharing (--kinship --ibdseg options, respectively). We also considered the option --unrelated to define a maximal set of unrelated individual (up to third-degree relationships).

### Demographic Inference

#### f-Statistics-Based Tests and Admixture Graph Construction

*f*-statistics based demographic inference (Patterson et al. 2012) were carried out with the R package *poolfstat* v2.2.0 (Gautier et al. 2022). We used the compute.fstats function to estimate the different *f*-statistics including $F_{3}$ for all the population triplets and $F_{4}$ for all population quadruplets. As for the Wright fixation indexes previously described, SE of the estimated statistics (and their corresponding *Z*-scores for $\;f_{3}$) were estimated using block-jackknife defining blocks of 250 consecutive SNPs (i.e. option nsnp.per.bjack.block=250). Following Patterson et al. (2012), formal tests of population admixture were carried out using the estimated $\;f_{3}$ statistics, a negative *Z*-score (Z<−1.65 at the 95% significance threshold) associated to an $\;f_{3}$ for a given population triplet A;B,C indicating that the target population A is admixed between two source populations each related to B and C. Admixture graph construction and exploration were carried out with *poolfstat* utilities using a semiautomatic approach similar to that described in Gautier et al. (2022). We relied in particular on the graph.builder function (ran with default options) to position populations onto scaffold graphs. The fit of the best-fitting graphs (based on the BIC criterion) was further validated with the *compare.fitted.fstats* function to compare to which extent the estimated *f*-statistics depart from their predicted values based on the fitted admixture graph parameters via a *Z*-score (Patterson et al. 2012; Lipson 2020; Gautier et al. 2022). The main steps of the graph construction are illustrated and detailed in supplementary fig. S5, Supplementary Material online. Based on the corresponding inferred history and as detailed in supplementary fig. S6, Supplementary Material online, the EUT populations represented in the W50K were ranked for their proximity with the European ancestral source of the TAF or MOK using $\;f_{3}$ and $\;f_{4}$ estimates for all (X;ZMA,Y) and (Y,NDA;X,GIR) configurations, respectively (where X=TAF or X=MOK and Y is the tested EUT population). Finally, the 95% CI of the proportion of EUT ancestry was (re)estimated more accurately for TAF (and MOK) using $F_{4}$-ratios as described supplementary fig. S6, Supplementary Material online with the *compute.f4ratio**poolfstat* function (Patterson et al. 2012; Gautier et al. 2022).

#### Estimation of the Timing of Admixture

We estimated the timing of admixture events (in generations) with the program ALDER v(1.03) (Loh et al. 2013). This approach relies on the modeling of the exponential decay of admixture-induced LD in a target admixed population as a function of genetic distance, using a LD measure weighted by AFs in either one or a pair of source population proxies. Genetic distances between pairs of SNPs were derived from physical distances assuming a cM to Mb ratio of 1 and a 6-year generation time was assumed to convert the timing from generations to years (Magnier et al. 2022).

#### Inference of the Recent Population Size History

Historical effective population sizes ($N_{e}$) were inferred with the program GONE that implements the approach developed by Santiago et al. (2020) to fit the observed spectrum of LD of pairs of loci over a wide range of recombination rates (which we derived from physical map distances assuming a cM to Mb ratio equal to 1 as above). Following Magnier et al. (2022), we adopted a block-jackknife approach to estimate CIs for the inferred $N_{e}$ trajectories by defining nonoverlapping blocks of 250 consecutive SNPs as above (block size of ca. 15 Mb).

#### Age-Based Partitioning of Individual Inbreeding

To identify and classify HBD segments, we relied on the model-based approach that is implemented in the R package RZooRoH (v0.3.2.1) (Druet and Gautier 2017, 2022; Bertrand et al. 2019). Within individual genomes, HBD segments correspond to segments inherited twice from a common ancestor as a result of inbreeding, and are often detected as runs-of-homozygosity (ROH), which are used as a proxy for HBD. In the ZooRoH model, autozygosity is partitioned into multiple HBD classes. HBD classes are defined by their rate parameter $R_{c}$, which determines the expected length of HBD segments, and correspond to groups of ancestors present in distinct past generations. To do this, we converted the VCF into a genotype probabilities (GPs) file (GEN format) using bcftools (v1.10.2) and the Phred-likelihood (PL) field, retaining only the 1,091,824 SNPs included in commercial genotyping arrays (Nicolazzi et al. 2014) and fitted the “layer” model (Druet and Gautier 2022) specifying 13 HBD classes with rates $R_{c}$ equal to {2;4;8;…;8,192} .

### Estimation of Genetic Diversity from WGS Data

Genetic heterozygosities based on WGS data were estimated from each individual genome alignments (bam) files described above that were further filtered for mapping quality and duplicate reads with the program *view* (run with option -q 20) and *rmdup* of the *samtools* (v1.13) suite (Li et al. 2009). To that end we relied on the program *mlrho* version 2.8 (Haubold et al. 2010) that implements a maximum-likelihood estimator of the population mutation rate ($\theta =4N_{e}\mu$) which fairly approximates heterozygosity under an infinite sites model (and providing *θ* is small), while simultaneously estimating sequencing error rates and accounting for binomial sampling of parental alleles (Lynch 2008). Following Gautier et al. (2016), only sites covered by 3 to 30 reads (after discarding bases with a base quality BAQ<25) were retained in the computation.

For further analyses of genetic diversity and genetic load, the VCF was recalibrated using the GATK ’s VariantRecalibrator command (VQSR step) using the file that was used for BQSR as known set and a file with 1,213,314 SNPs from commercial arrays as the truth set (Nicolazzi et al. 2014). We then selected bi-allelic SNPs with a quality score corresponding to 99% conservation of the truth set and that mapped to autosomes. In addition, SNPs called for less than 90% of individuals or with an average individual depth of coverage (DP) <5 or ≥20 were discarded, leaving 23,383,523 SNPs for further analyses.

AFs were estimated per population from individual genotype likelihoods (PL) using a custom implementation (in modern Fortran) of the EM-algorithm described by Kim et al. (2011). The number of segregating sites per population was estimated as the number of sites with a 0.001<AF<0.999. The number of fixed sites per population was derived from the proportion of sites with a frequency of the derived allele greater than 0.999. For allele polarization, we repeated the variant calling by adding the BAM files of one individual from four outgroups (*Bison bison*, *Bison bonasus*, *Bos javanicus*, and *Bos gaurus*) (supplementary table S2, Supplementary Material online). When all outgroup individuals were homozygous for an identical allele, and at most one outgroup had a missing genotype, we called this allele the ancestral allele. Using this approach we were able to define the ancestral allele for 83.8% of variants. The number of fixed sites per population was then estimated as the proportion of fixed derived alleles multiplied by the total number of sites. To compare the SFS, we calculated the probability of the presence of 1 to 15 derived alleles in each population to have only discrete values. This was, therefore, applied only to variants for which the derived allele was identified and without missing genotypes in that population. These genotype probabilities (GP) were derived from the PL of the eight individuals from the population and by enumerating the 6,561 possible genotype combinations (three possible genotypes for eight individuals). For each combination, the number of derived alleles is known and the probability can be estimated as the product of GP values. Finally, the probability of the presence of N derived alleles in the population was estimated as the sum of probabilities of all combinations with N derived alleles.

### Genetic Load and Distribution of Deleterious Variants

We relied on the *variant effect predictor* (VEP) v95.0 program (McLaren et al. 2016) to annotate our variants. Variants annotated as “stop gained,” “splice donor,” or “splice acceptor” were defined as LoF variants. Additional classes consisted of “intergenic,” “synonymous,” (S) and “nonsynonymous” (NS) variants, which could be further subdivided into “deleterious NS” and “tolerated NS” classes. In the case of multiple annotations, we retained the most severe annotation. This classification of variants was first used to compare, across the different populations, the proportions of segregating and fixed variants in different classes and the SFS obtained for each class. We then calculated measures related to the relaxation of purifying selection, the occurrence of purging of deleterious alleles and to the masked and realized loads. First, we estimated the NS/S ratio as the ratio of heterozygous genotypes for these two categories per individual (Cruz et al. 2008; Renaut and Rieseberg 2015). Next, the masked and realized load, measured for a group of deleterious variants, were estimated as in Smeds and Ellegren (2023). The masked load of an individual is defined as the proportion of heterozygotes genotypes, whereas the realized load is the proportion of genotypes homozygous for the derived allele (both statistics were calculated using only the called genotypes of that category within each individual). These first measures using genotype counts per individual were calculated using GPs. Finally, we calculated the relative number of derived alleles $R_{X/Y}$ (Do et al. 2015) in two populations *X* and *Y*. To do this, the probability of sampling a derived allele in population *X* and not in population *Y* is calculated for each SNP using the estimated AF of the derived alleles in population *X* ($f_{X}$) and *Y* ($f_{Y}$) as ($f_{X}(1-f_{Y})$) and then summed over all SNPs. The opposite probabilities, ($f_{Y}(1-f_{X})$), are summed over all SNPs and $R_{X/Y}$ is finally obtained as the ratio of these two values. We estimated $R_{X/Y}$ for missense and LoF variants, and these values were standardized by the values obtained for intergenic variants (as proxy for neutral variants). This standardization corrects for differences in branch lengths (Do et al. 2015). Note that we obtained $R_{X/Y}$ values close to 1 for intergenic variants, indicating that standardization was not necessary. The CI of the $R_{X/Y}$ values was obtained by a block jackknife procedure as in Xue et al. (2015). Briefly, we divided the genome into 100 blocks of consecutive SNPs, with the same number of SNPs per block, and repeated the calculation of $R_{X/Y}$ by ignoring each block one by one.

### Adaptive History

#### Estimation of Genetic Offset

The relative degree of “maladaption” to the environment of Amsterdam island was evaluated using a GO statistic (Gain et al. 2023) for all the cattle populations represented in the W50K data set. This statistic quantifies the difference between a source and a target environment as a weighted distance between their underlying environmental (e.g. bioclimatic) covariable values. The weights on the covariables are directly related to the importance of their association with the (adaptive) genomic composition of the breeds that is estimated under a GEA model assuming the different populations are adapted to their environment of origin. Following Gain et al. (2023), we relied on a linear modeling of the relationship between the genetic diversity of the 32 cattle population in the W50K data set and 19 bioclimatic covariables (averaged values over the period 1981 to 2010 at a 30 arc sec resolution) that were extracted from the CHELSA (v2.1, accessed the 2023 May 21) database (Karger et al. 2017, 2018) to characterize their environment based on the GPS coordinates of their birthplace (supplementary table S2, Supplementary Material online). More precisely, we first carried out a PCA on the scaled and centered covariables using the R package *ade4* (Dray and Dufour 2007), and retained the first seven PCs that together explained 98.7% of the overall variation. We then fitted a linear model using the *covmcmc* model implemented in BayPass v2.4 (Gautier 2015) and used the resulting (posterior mean) estimates of the vectors $\beta_{k}$ of the $n_{\mathrm{snp}}=40,426$ SNP regression coefficients for each of the seven (scaled) PCs *k*. In short, the regression coefficients summarize the effect of each environmental covariable on the distribution of AFs corrected for the neutral structuring of genetic diversity. The GO for each population *j* environment with respect to the Amsterdam island environment characterized by the vectors $e_{\;j}$ and $e_{taf}$ of seven PCs, respectively, was then computed as


$$GO_{j}=\frac{1}{n_{\mathrm{snp}}}(e_{\;j}-e_{taf}{)}^{\prime }B^{\prime }B(e_{\;j}-e_{taf})$$

where B is the ($n_{\mathrm{snp}}\times 7$) matrix with all the seven $\beta_{k}$ vectors stacked side by side (Gain et al. 2023). As a matter of comparison we also computed the (unweighted) environmental distance as $\delta_{j}=\sqrt{\left(1/n_{e})(e_{\;j}-e_{taf}{)}^{\prime }(e_{\;j}-e_{taf}\right)}$ that simply corresponds to the Euclidean distance between environmental PCs (where $n_{e}=7$ is the number of environmental PCs). It should be noted that we have computed GO here assuming that the sample of bovine populations analyzed, representative of worldwide diversity but with a deliberate focus on EUT breeds, are adapted to the environment of their birthplace of origin (in the sense that adaptive alleles are at their local optimum frequency), while also including the TAF population to allow the representation of the Amsterdam island conditions. This is reasonable since the primary goal of the GO computation is to model the relationship between environmental conditions (characterized by a set of 19 bioclimatic covariates each averaged over the period 1981 to 2010) and the structuring of genetic diversity in the underlying populations via a GEA model. This allows environmental distances to be appropriately weighted to account for genetic adaptation in the derivation of GO (Gain et al. 2023). To compute the GO distribution over the whole European continent, we extracted the 19 environmental bioclimatic covariates for all positions across the entire European continent from CHELSA v2.1. These were then transformed into PCs using the above PCA loadings (with the *suprow* function of *ade4*) that we rescaled as the breed bioclimatic PCs to compute GO. The resulting GO rasters were processed and analyzed at a resolution of 0.01^∘^ with utilities from the raster R package (Hijmans 2022).

#### Genomic Prediction of Breeding Values for Height Based on WGS Data

To investigate whether the TAF are enriched in alleles associated with short stature as a result of possible selection, we compared the frequency of such alleles in eight sequenced individuals with the frequency observed in the four other breed samples from the WGS data set. To do this, we used a list of 164 variants identified in a large cattle meta-analysis (Bouwman et al. 2018) (mapped to the UMD3.1 bovine reference genome). Among them, 105 variants, with matching reference alleles in both reference genomes, were found polymorphic in our data. Individual genotypes for these alleles were then used to predict height differences between individuals, as the sum over all SNPs of the allele dosage by the reported allele effect on height. This is equivalent to a comparison allele frequencies, weighted by their effect as precisely estimated by Bouwman et al. (2018) on various cattle breeds.

#### Genome Scan with EHH-Based Test Using 530K SNP Haplotypes

To increase the power and sensitivity of the detection of footprints of selection, we first built a data set consisting of 71 individuals (18 TAF, 23 ZMA, and 30 JER) that were genotyped, either directly or via imputation, for 530,769 autosomal SNPs from the Illumina BovineHD SNP assay (comprising >770,000 SNPs). To that end, we first extracted genotypes at 718,718 BovineHD SNPs that we could unambiguously identify in the vcf file we generated with our WGS data and recoded them in the so-called “Top” format of the chip. We then combined the obtained genotypes for the eight TAF, eight ZMA, eight MAY, and eight JER sequenced individuals with SNP genotyping data for the 18 TAF on the BovineSNP50K assay and for the 30 JER, 32 MAY, and 26 ZMA individuals on the BovineHD assay that were publicly available from previous studies (Magnier et al. 2022) and were extracted from the Widde database (Sempéré et al. 2015). We used genotypes from 24 the individuals (eight TAF, eight MAY, and eight ZMA) that were both sequenced and genotyped to assess the genotype concordance rate. Among their 8,843,201 genotypes available from both WGS data (with a GQ > 20) and the genotyping array, only 0.42% were found to be different. For those, we chose to keep the SNP assay genotype in the combined data set further excluding five individuals (three ZMA and two MAY) that remained poorly genotyped. Likewise, we only retained the 547,214 SNPs that were genotyped on more than 90% of the individuals in each of the four population samples. We then proceeded with the imputation of missing genotypes for the ten TAF individuals that were only genotyped on the 50K SNP assay (corresponding to the targets). The reference panel consisted of the 40 JER, the 30 MAY, the 8 TAF, and the 23 ZMA individuals genotyped for the 530,769 SNPs. We first carried out haplotype phasing of the 29 bovine autosomes with Beagle v5.4 (Browning et al. 2021) for the reference and target panels separately. We then carried out missing genotypes imputation using Beagle v5.4 (Browning et al. 2018).

Based on the resulting haplotypes, we then carried out genome-wide scan for footprints of positive selection using EHH-based tests within the TAF population using the iHS (Voight et al. 2006) statistic for within-population and the Rsb statistic (Tang et al. 2007) for pairwise-population analyses, as implemented in the R package *rehh* v3.1.2 (Gautier et al. 2017; Klassmann and Gautier 2022). For the standardization of iHS, we only retained the 204,867 SNPs with a MAF>0.01 and the alleles were not polarized (i.e. *scan_hh* was run with polarized=FALSE and *ihh2ihs* was run with options min_maf=0.01 and freqbin=1) as discussed in Klassmann and Gautier (2022) and section 7.6 of the online *rehh* vignette. The iHS statistics was further transformed into ${\text{p}}_{\mathrm{iHS}}=-\log_{10}\;(1-2|\Phi (\text{iHS})-0.5|)$ (where Φ(x) is the Gaussian cumulative function). Assuming iHS is normally distributed under neutrality, the resulting $p_{\mathrm{iHS}}$ may then be interpreted as two-sided a *P*-value (on a negative $\log_{10}$ scale) associated with the neutral hypothesis of no selection. For the analyses based on Rsb derived from the ratio of TAF integrated site EHH in the numerator and either that of JER or ZMA in the denominator, we instead focused on one-sided *P*-value of the form ${\text{p}}_{\mathrm{Rsb}}=-\log_{10}\;(1-\Phi (\text{Rsb}))$ to identify TAF-specific signals only. Candidate regions were identified by combining the obtained SNP-specific *P*-values into a local-score derived from a Lindley process as described in Fariello et al. (2017) with a SNP score equal to $-\log_{10}\;(p)-\xi$. We here chose ξ=2 and only retained the windows with a local-score peak value significant at a 1% *P*-value threshold (analytically derived for each chromosome separately). The obtained windows were then annotated for their number of SNPs, their size, the position of the iHS or Rsb peak, and the closest gene using the NCBI annotation gff file for the ARS-UCD1.2 genome assembly.

The candidate genes were functionally annotated using IPA software (Ingenuity Systems, Inc. 2023) considering the Ingenuity Pathway Knowledge Base (IPKB) as reference set. The top significant functions and diseases (P-value<0.05) were obtained by comparing functions associated with the candidate genes under selection against functions associated with all genes in the reference set, using the right-tailed Fisher exact test.

## Supplementary Material

msae121_Supplementary_Data

## Data Availability

All SNP genotyping data, including those newly generated for the TAF and MOK individuals, are publicly available from the WIDDE repository (http://widde.toulouse.inra.fr/widde/). WGS data, including those newly generated for the 8 TAF individuals, are available from the NCBI SRA repository (see Supplementary Table S3, Supplementary Material online, for details of accession run IDs).
