# Supplementary material for: Genomic Reconstruction of the Successful Establishment of a Feralized Bovine Population on the Subantarctic Island of Amsterdam
Source: Mol Biol Evol. 2024 Jun 18;41(7):msae121. doi: 10.1093/molbev/msae121 (PMC11339654; doi:10.1093/molbev/msae121)
Supplement: msae121_Supplementary_Data [file msae121_supplementary_data.pdf]

ID	Collection Year	Sex	Estimated Birth year	Genotyping	Whole Genome Sequence
TAF_5610	1992/03	Female	1984	BovineSNP50K	n.a.
TAF_5614	1992/03	Female	1984	BovineSNP50K	n.a.
TAF_5603	1992/03	Female	1987	BovineSNP50K	SRR25784578
TAF_5604	1992/03	Female	1989	BovineSNP50K	SRR25784577
TAF_5608	1992/03	Female	1989	BovineSNP50K	n.a.
TAF_5609	1992/03	Female	1985	BovineSNP50K	SRR25784566
TAF_5612	1992/03	Female	1989	BovineSNP50K	n.a.
TAF_5613	1992/03	Female	1990	BovineSNP50K	SRR25784561
TAF_5617	1992/03	Female	1986	BovineSNP50K	SRR25784560
TAF_5618	1992/03	Female	1985	BovineSNP50K	SRR25784559
TAF_5619	1992/03	Female	1985	BovineSNP50K	SRR25784558
TAF_5622	1992/03	Female	1987	BovineSNP50K	SRR25784557
TAF_MN279	2006/03/29	Male	2004	BovineSNP50K	n.a.
TAF_MN280	2006/03/29	Male	2000	BovineSNP50K	n.a.
TAF_MN281	2006/03/31	Female	1999	BovineSNP50K	n.a.
TAF_MN282	2006/03/31	Male	2004	BovineSNP50K	n.a.
TAF_MN284	2006/04/05	Female	1999	BovineSNP50K	n.a.
TAF_MN285	2006/04/05	Female	2003	BovineSNP50K	n.a.

Table S1: Description of the individual sample from the bovine population of the Amsterdam island. Birth year were approximated from individual dental age estimation. All genotyped data (including the new ones) are available from the WIDDE repository (Sempéré *et al.*, 2015) and newly generated whole genome sequence data are publicly available in the NCBI SRA repository (PRJNA1010533 project).

Code	Breed Name	Origin (Type)	Sampling Country	Long.;Lat. (ref. env.)	n_{ind} (50K)	n_{ind} (WGS)
ABO	Abondance	France (EUT)	France	6.723 ; 46.28	22 (Gautier *et al.*, 2010)	-
AUB	Aubrac	France (EUT)	France	2.848 ; 44.37	22 (Gautier *et al.*, 2010)	-
BPN	Bretonne Black Pied France (EUT)	France (EUT)	France	-2.980 ; 48.07	18 (Gautier *et al.*, 2010)	-
BSW	Brown Swiss	Switzerland (EUT)	USA	8.658 ; 47.02	24 (Matukumalli *et al.*, 2009)	-
CHA	Charolais	France (EUT)	France	4.275 ; 46.43	20 (Gautier *et al.*, 2010)	-
GAS	Gascon	France (EUT)	France	1.638 ; 43.16	22 (Gautier *et al.*, 2010)	-
GIR	Gir	India (ZEB)	Brazil	-51.93 ; -14.26	24 (Matukumalli *et al.*, 2009)	-
GNS	Guernsey	Channel Islands (EUT)	USA, UK	-2.533 ; 49.45	21 (Matukumalli *et al.*, 2009)	-
HFD	Hereford	England (EUT)	USA, NZL	-2.708 ; 52.06	31 (Matukumalli *et al.*, 2009)	-
HOL	Holstein (FR)	Netherlands (EUT)	France, NZL	10.13 ; 54.33	30 (Gautier *et al.*, 2010)	8 (Daetwyler *et al.*, 2014)
JER	Jersey (US)	Channel Islands (EUT)	USA, NZL	-2.117 ; 49.20	28 (Matukumalli *et al.*, 2009)	8 (Daetwyler *et al.*, 2014)
JE2	Jersey (FR)	Channel Islands (EUT)	France	-2.117 ; 49.20	21 (Gautier *et al.*, 2010)	-
LAG	Lagune	Benin (AFT)	Benin	2.316 ; 9.308	44 (Gautier *et al.*, 2009)	-
LMS	Limousine	France (EUT)	USA	1.275 ; 45.20	44 (Gautier *et al.*, 2010)	-
MAN	Maine-Anjou (*a.k.a.* Rouge des Prés)	France (EUT)	France	-0.7028 ; 47.83	46 (Gautier *et al.*, 2010)	-
MAR	Maraichine (*a.k.a.* Parthenaise)	France (EUT)	France	-1.084 ; 46.42	19 (Gautier *et al.*, 2010)	-
MAY	Zebu from Mayotte	Mayotte Island (AFZ)	France	45.23 ; -12.78	30 (Magnier *et al.*, 2022)	**8**
MOK	Moka	La Réunion Island (HYB)	France	55.54 ; -21.12	**31**	-
MON	Montbeliarde	France (EUT)	France	5.876 ; 47.19	30 (Gautier *et al.*, 2010)	-
NDA	N'Dama	West-Africa (AFT)	Guinea	-13.70 ; 9.517	25 (Matukumalli *et al.*, 2009)	-
NEL	Nelore	India (ZEB)	Brazil	-38.51 ; -12.97	21 (Matukumalli *et al.*, 2009)	-
NOR	Normande	France (EUT)	France	-0.3253 ; 49.20	30 (Gautier *et al.*, 2010)	-
PMT	Piedmontese	Italy (EUT)	Italy	7.667 ; 45.07	24 (Matukumalli *et al.*, 2009)	-
RDB	Raço di Bioù	France (EUT)	France	4.138 ; 43.54	29 (Flori *et al.*, 2019)	-
SAL	Salers	France (EUT)	France	2.495 ; 45.14	22 (Gautier *et al.*, 2010)	-
SHK	Sheko	East-Africa (AFZ)	Ethiopia	40.49 ; 9.145	20 (Matukumalli *et al.*, 2009)	-
SOM	Somba	Togo (AFT)	Togo	2.605 ; 6.497	44 (Gautier *et al.*, 2009)	-
TAF	Cattle of Amsterdam Island	Amsterdam Island (HYB)	France	77.55 ; -37.83	**18**	**8**
TAR	Tarine	France (EUT)	France	5.953 ; 45.54	18 (Gautier *et al.*, 2010)	-
VOS	Vosgienne	France (EUT)	France	7.386 ; 48.01	20 (Gautier *et al.*, 2010)	-
ZFU	Zebu Fulani	West-Africa (AFZ)	Benin	9.132 ; 10.93	43 (Gautier *et al.*, 2009)	-
ZMA	Zebu from Madagascar	Madagascar (AFZ)	Madagascar	47.53 ; -18.91	35 (Gautier *et al.*, 2009)	**8**

Table S2: Sample description with country of origin and population type (i.e. EUT=European Taurines, AFT=African Taurines, AFZ=African Zebus, ZEB=Zebus of Indian origin, or HYB=Hybrids); the country of sampling; the GPS coordinate (in decimal degrees) used to retrieve environmental covariates; the number of individuals genotyped on the BovineSNP50 Illumina assay and included in the W50K dataset; and the number of individuals sequenced (WGS) with associated reference. GPS coordinates specifying the environment of each population were taken as the birthplace of the breed including for GIR and NEL in Brazil (Gautier *et al.*, 2010). Individuals from the Jersey (EUT) breed were divided into two distinct groups corresponding to i) 21 individuals (coded JE2) sampled in northwestern France (Maine-et-Loir county) and born between 1982 and 1990 (Gautier *et al.*, 2010); and ii) 28 individuals (coded JER) sampled in the USA (Matukumalli *et al.*, 2009). The newly genotyped (n=49) and sequenced (n=24) individuals are highlighted in bold. All genotyped data (including the new ones) are available from the WIDDE repository (Sempéré *et al.*, 2015) and the newly generated whole genome sequence data are publicly available in the NCBI SRA repository (project PRJNA1010533).

Pop.	Type	% SNP mono.	Heterozygosity	F_{IS}
TAF		43.3	0.216 [0.212;0.222]	-0.019 [-0.034;-0.004]
MOK		12.4	0.263 [0.259;0.267]	0.022 [0.016;0.030]
LAG	AFT	41.0	0.190 [0.187;0.193]	0.041 [0.034;0.046]
NDA	AFT	34.4	0.214 [0.211;0.216]	0.009 [0.005;0.014]
SOM	AFT	23.5	0.231 [0.228;0.234]	0.041 [0.037;0.046]
MAY	AFZ	34.0	0.204 [0.200;0.209]	0.037 [0.033;0.044]
SHK	AFZ	23.3	0.255 [0.251;0.258]	-0.005 [-0.010;-0.001]
ZFU	AFZ	19.5	0.247 [0.243;0.251]	0.003 [-0.001;0.006]
ZMA	AFZ	32.6	0.204 [0.199;0.209]	0.012 [0.006;0.017]
ABO	EUT	12.9	0.300 [0.298;0.303]	-0.039 [-0.044;-0.032]
AUB	EUT	10.9	0.307 [0.304;0.310]	0.011 [0.006;0.016]
BPN	EUT	11.4	0.315 [0.312;0.318]	-0.011 [-0.018;-0.004]
BSW	EUT	18.2	0.274 [0.268;0.277]	-0.040 [-0.048;-0.031]
CHA	EUT	9.79	0.318 [0.315;0.320]	0.002 [-0.004;0.008]
GAS	EUT	11.3	0.308 [0.305;0.311]	0.002 [-0.002;0.008]
GNS	EUT	17.7	0.283 [0.279;0.285]	0.020 [0.012;0.030]
HFD	EUT	9.55	0.323 [0.321;0.326]	0.064 [0.057;0.074]
HOL	EUT	9.98	0.316 [0.312;0.319]	-0.000 [-0.007;0.008]
JER	EUT	20.4	0.263 [0.258;0.266]	-0.009 [-0.017;0.001]
JE2	EUT	17.8	0.281 [0.276;0.284]	0.001 [-0.005;0.012]
LMS	EUT	7.41	0.314 [0.311;0.317]	0.004 [-0.000;0.008]
MAN	EUT	8.83	0.300 [0.295;0.303]	-0.013 [-0.018;-0.010]
MAR	EUT	10.1	0.318 [0.316;0.320]	-0.013 [-0.020;-0.007]
MON	EUT	13.5	0.291 [0.289;0.294]	-0.036 [-0.043;-0.029]
NOR	EUT	11.7	0.300 [0.297;0.303]	-0.032 [-0.037;-0.027]
PMT	EUT	8.34	0.321 [0.319;0.324]	-0.009 [-0.013;-0.006]
RDB	EUT	21.5	0.258 [0.254;0.261]	-0.029 [-0.039;-0.020]
SAL	EUT	13.3	0.294 [0.290;0.296]	0.018 [0.014;0.027]
TAR	EUT	14.4	0.300 [0.297;0.302]	-0.007 [-0.013;-0.000]
VOS	EUT	11.1	0.309 [0.306;0.311]	-0.012 [-0.019;-0.008]
GIR	ZEB	39.6	0.166 [0.162;0.172]	0.007 [0.001;0.015]
NEL	ZEB	40.8	0.164 [0.159;0.169]	-0.019 [-0.026;-0.013]

Table S3: Within population genetic diversity estimated on the W50K data set. The table report for each population i) the percentage of monomorphic SNPs; ii) the average heterozygosity estimated with `poolfstat` (Gautier *et al.*, 2022); and iii) the F_{IS}. For the two latter statistics 95% confidence intervals derived from the block-jackknife estimates of their standard errors are given into brackets. It is important to note that heterozygosity must be interpreted with cautious and are a poor estimator of genetic diversity particularly for ZEB, AFZ and AFT breeds because of the strong SNP ascertainment bias towards SNPs overrepresented in the EUT populations in the BovineSNP50K genotyping assay (Gautier *et al.*, 2010; Matukumalli *et al.*, 2009).

RGID	Breed Name	Sexe	SRA Run ID	Reference	Instrument	Mean Realized Coverage	
						Auto.	X
TAF_5603	AmsterdamIsland	Female	SRR25784578	thisstudy	HiSeqX	15.2	15.9
TAF_5604	AmsterdamIsland	Female	SRR25784577	thisstudy	HiSeqX	15.8	16.2
TAF_5609	AmsterdamIsland	Female	SRR25784566	thisstudy	HiSeqX	14.3	14.7
TAF_5613	AmsterdamIsland	Female	SRR25784561	thisstudy	HiSeqX	14.6	15.2
TAF_5617	AmsterdamIsland	Female	SRR25784560	thisstudy	HiSeqX	19.3	19.8
TAF_5618	AmsterdamIsland	Female	SRR25784559	thisstudy	HiSeqX	14.9	15.5
TAF_5619	AmsterdamIsland	Female	SRR25784558	thisstudy	HiSeqX	17.8	18.4
TAF_5622	AmsterdamIsland	Female	SRR25784557	thisstudy	HiSeqX	17.0	17.9
ZMA_1570	MadagascarZebu	Female	SRR25784556	thisstudy	HiSeq2500	6.04	6.21
ZMA_1583	MadagascarZebu	Female	SRR25784555	thisstudy	NovaSeq	23.8	23.4
ZMA_1585	MadagascarZebu	Female	SRR25784576	thisstudy	HiSeq2500	10.0	10.4
ZMA_1595	MadagascarZebu	Female	SRR25784575	thisstudy	HiSeq2500	8.00	8.42
ZMA_1649	MadagascarZebu	Female	SRR25784574	thisstudy	HiSeq2500	7.92	7.98
ZMA_1650	MadagascarZebu	Female	SRR25784573	thisstudy	NovaSeq	33.3	33.0
ZMA_1661	MadagascarZebu	Female	SRR25784572	thisstudy	NovaSeq	24.5	23.6
ZMA_1666	MadagascarZebu	Female	SRR25784571	thisstudy	HiSeq2500	9.16	9.54
MAY_011	MayotteZebu	Female	SRR25784570	thisstudy	NovaSeq	16.1	15.4
MAY_015	MayotteZebu	Female	SRR25784569	thisstudy	NovaSeq	16.0	15.2
MAY_021	MayotteZebu	Male	SRR25784568	thisstudy	NovaSeq	17.3	8.86
MAY_071	MayotteZebu	Male	SRR25784567	thisstudy	NovaSeq	14.0	7.55
MAY_095	MayotteZebu	Male	SRR25784565	thisstudy	NovaSeq	18.6	9.38
MAY_175	MayotteZebu	Male	SRR25784564	thisstudy	NovaSeq	17.7	8.69
MAY_219	MayotteZebu	Female	SRR25784563	thisstudy	NovaSeq	13.9	14.0
MAY_371	MayotteZebu	Female	SRR25784562	thisstudy	NovaSeq	17.5	16.6
HOL_0007	Holstein	Male	SRR1262661	(Daetwyler *et al.*, 2014)	HiSeq2000	12.1	2.75
HOL_0018	Holstein	Male	SRR1262672	(Daetwyler *et al.*, 2014)	HiSeq2000	11.7	6.10
HOL_0025	Holstein	Male	SRR1262679	(Daetwyler *et al.*, 2014)	HiSeq2000	11.3	6.00
HOL_0064	Holstein	Male	SRR1262718	(Daetwyler *et al.*, 2014)	HiSeq2000	11.0	5.93
HOL_0083	Holstein	Male	SRR1262737	(Daetwyler *et al.*, 2014)	HiSeq2000	10.6	4.92
HOL_0117	Holstein	Male	SRR1262771	(Daetwyler *et al.*, 2014)	HiSeq2000	8.58	4.85
HOL_0123	Holstein	Male	SRR1262777	(Daetwyler *et al.*, 2014)	HiSeq2000	8.32	4.76
HOL_0127	Holstein	Male	SRR1262781	(Daetwyler *et al.*, 2014)	HiSeq2000	11.6	3.73
JER_0003	Jersey	Male	SRR1262791	(Daetwyler *et al.*, 2014)	HiSeq2000	8.35	3.91
JER_0005	Jersey	Male	SRR1262793	(Daetwyler *et al.*, 2014)	HiSeq2000	11.9	5.76
JER_0009	Jersey	Male	SRR1262797	(Daetwyler *et al.*, 2014)	HiSeq2000	9.21	5.36
JER_0010	Jersey	Male	SRR1262798	(Daetwyler *et al.*, 2014)	HiSeq2000	10.5	2.78
JER_0011	Jersey	Male	SRR1262799	(Daetwyler *et al.*, 2014)	HiSeq2000	10.5	3.98
JER_0013	Jersey	Male	SRR1262801	(Daetwyler *et al.*, 2014)	HiSeq2000	11.6	2.33
JER_0014	Jersey	Male	SRR1262802	(Daetwyler *et al.*, 2014)	HiSeq2000	10.3	4.68
JER_0015	Jersey	Male	SRR1262803	(Daetwyler *et al.*, 2014)	HiSeq2000	10.6	5.79
BBI_001	Bison bison	U	SRR6448737→740	(Wu *et al.*, 2019)	HiSeq2000	25.9	30.7
BBO_011	Bison bonasus	U	SRR6448682→684,SRR6448670	(Wu *et al.*, 2019)	HiSeq2000	29.5	29.5
BGA_001	Bos gaurus	U	SRR6448732→735	(Wu *et al.*, 2019)	HiSeq2000	17.0	16.6
BJA_006	Bos javanicus	U	SRR6448720	(Wu *et al.*, 2019)	HiSeq2000	16.9	9.26

Table S4: Description of the whole genome sequences, including the 24 newly generated ones, used in the study. Mean coverage for autosomes and X chromosome were estimated with *Samtools* (v1.9) *stats* program (Li *et al.*, 2009) after read duplicates removal.

f_3 triplet	Estimate (x10^3)	95% Confidence Interval
TAF;ZMA,JER	46.35	[43.30;49.19]
TAF;ZMA,JE2	46.43	[43.40;49.32]
TAF;ZMA,HFD	46.41	[43.68;49.25]
TAF;ZMA,BPN	46.70	[43.84;49.35]
TAF;ZMA,AUB	47.26	[44.26;49.83]
TAF;ZMA,MAR	47.25	[44.24;50.05]
TAF;ZMA,SAL	47.44	[44.35;50.17]
TAF;ZMA,MAN	47.41	[44.47;50.15]
TAF;ZMA,GNS	47.31	[44.44;50.28]
TAF;ZMA,CHA	47.65	[44.78;50.34]
TAF;ZMA,NOR	47.66	[44.67;50.53]
TAF;ZMA,TAR	47.93	[44.77;50.46]
TAF;ZMA,LMS	47.85	[44.88;50.43]
TAF;ZMA,GAS	48.24	[45.10;50.77]
TAF;ZMA,HOL	48.11	[45.01;50.86]
TAF;ZMA,PMT	48.17	[45.21;50.76]
TAF;ZMA,VOS	48.54	[45.42;51.14]
TAF;ZMA,BSW	48.48	[45.40;51.32]
TAF;ZMA,ABO	48.49	[45.62;51.30]
TAF;ZMA,MON	48.70	[45.59;51.41]
TAF;ZMA,RDB	48.71	[45.68;51.77]

Table S5: Estimated F_3 values in increasing order for all (TAF;ZMA,EUT) configurations where. The EUT population the closest to the original European source population of the TAF is expected to display the lowest value (see Figure S6).

f_4 quadruplet	Estimate (x10^3)	95% Confidence Interval
JE2,NDA;TAF,GIR	14.28	[12.33;16.11]
BPN,NDA;TAF,GIR	14.04	[12.33;15.90]
AUB,NDA;TAF,GIR	13.98	[12.38;15.83]
JER,NDA;TAF,GIR	14.15	[12.16;16.00]
SAL,NDA;TAF,GIR	13.82	[12.30;15.59]
MAR,NDA;TAF,GIR	13.88	[12.23;15.53]
GNS,NDA;TAF,GIR	13.78	[11.98;15.48]
HFD,NDA;TAF,GIR	13.76	[11.86;15.44]
NOR,NDA;TAF,GIR	13.59	[11.94;15.33]
MAN,NDA;TAF,GIR	13.60	[11.81;15.44]
TAR,NDA;TAF,GIR	13.16	[11.73;15.09]
CHA,NDA;TAF,GIR	13.36	[11.69;15.09]
LMS,NDA;TAF,GIR	13.18	[11.59;14.98]
GAS,NDA;TAF,GIR	12.85	[11.41;14.74]
PMT,NDA;TAF,GIR	12.91	[11.42;14.61]
RDB,NDA;TAF,GIR	13.01	[11.20;14.59]
HOL,NDA;TAF,GIR	12.61	[11.02;14.45]
VOS,NDA;TAF,GIR	12.53	[11.04;14.38]
ABO,NDA;TAF,GIR	12.69	[10.92;14.50]
MON,NDA;TAF,GIR	12.50	[10.84;14.44]
BSW,NDA;TAF,GIR	12.42	[10.49;14.33]

Table S6: F_4 estimated values for all (EUT,NDA;TAF,GIR) configurations where NDA and GIR are representative of AFT and ZEB ancestry. The EUT population the closest to the original European source population of the TAF is expected to display the highest value (see Figure S6).

Class	TAF	HOL	JER	ZMA	MAY
S	38,024 (0.42;1.00)	34,943 (0.41;0.98)	30,527 (0.41;0.98)	67,176 (0.41;0.98)	69,283 (0.41 ; 0.99)
NS	23,590 (0.26;1.00)	24,523 (0.29;1.10)	21,483 (0.29;1.11)	40,266 (0.25;0.95)	41,245 (0.25;0.95)
Tol. NS	17,796 (0.2;1.00)	18,101 (0.21;1.08)	15,796 (0.21;1.08)	29,801 (0.18;0.93)	30,460 (0.18;0.93)
Del. NS	4,896 (0.05;1.00)	5,636 (0.07;1.22)	4,960 (0.07;1.23)	8,896 (0.05;1.01)	9,160 (0.05;1.01)
LoF	524 (0.01;1.00)	538 (0.01;1.09)	552 (0.01;1.28)	996 (0.01;1.06)	1,014 (0.01;1.05)
ALL	9,036,293	8,520,472	7,440,903	16,223,315	16,706,828

Table S7: Number of segregating sites observed in the different populations in WGS data. The table gives the total number of variant sites and for each functional classes (S=Synonymous; NS=Non-Synonymous; Tol. NS= Tolerated NS; Del NS=Deleterious NS; and LoF=Loss-of-Function). For the four functional classes, the percentage with respect to the overall number of sites and the proportions relative to that observed in TAF population are given in parentheses.

Class	TAF	HOL	JER	ZMA	MAY
S	8,882 (0.41;1.00)	11,994 (0.44;1.07)	12,807 (0.44;1.05)	2,078 (0.41;1.00)	1,765 (0.39;0.94)
NS	4,505 (0.21;1.00)	5,962 (0.22;1.05)	6,402 (0.22;1.04)	1,126 (0.22;1.07)	1,041 (0.23;1.09)
Tol. NS	3,895 (0.18;1.00)	5,209 (0.19;1.06)	5,598 (0.19;1.05)	910 (0.18;1.00)	864 (0.19;1.05)
Del. NS	399 (0.02;1.00)	454 (0.02;0.90)	497 (0.02;0.91)	152 (0.03;1.63)	129 (0.03;1.53)
LoF	104 (0.005;1.00)	132 (0.005;1.00)	135 (0.005;0.95)	15 (0.003;0.62)	15 (0.003;0.68)
ALL	2,150,813	2,719,983	2,939,594	504,199	455,553

Table S8: Number of fixed sites observed in the different populations in WGS data. Sites with non-polarized variants or found monomorphic over all samples were discarded from the counts. The table gives the total number of fixed sites and for each functional classes (S=Synonymous; NS=Non-Synonymous; Tol. NS= Tolerated NS; Del NS=Deleterious NS; and LoF=Loss-of-Function). For the five functional classes, the percentage with respect to the overall number of sites and the proportions relative to that observed in TAF population are given parentheses.

uni. Rsb Test	Win. Pos. (chr:start-end in kb)	Win Size in kb (Nsnp)	Peak Pos. in kb (Stat. Value)	Nearest Gene (Pos. in kb ; dist. from peak)
JER/ZMA	03:49,466-49,768	302.5 (110)	49,482 (2.86)	ABCA4 (49,387-49,531 ; 0)
JER/ZMA⋆	06:85,793-88,358	2,564.6 (568)	86,797 (4.39)	SLC4A4 (86,449-86,813 ; 0)
JER/ZMA	06:68,561-69,197	636.7 (221)	68,750 (3.96)	SCFD2 (68,539-68,936 ; 0)
JER/ZMA	06:88,952-89,553	600.6 (168)	88,975 (3.5)	LOC100847175 (88,955-88,963 ; 12)
JER/ZMA	07:46,931-48,975	2,044 (541)	47,277 (4.48)	SLC25A48 (47,258-47,310 ; 0)
JER/ZMA	07:27,021-27,474	453.2 (103)	27,056 (3.81)	MARCHF3 (26,948-27,104 ; 0)
JER/ZMA	18:8,977-9,993	1,016 (344)	9,074 (5.32)	CDH13 (9,138-10,154 ; 64)
JER/ZMA⋆	20:24,413-27,203	2,790 (558)	26,070 (3.68)	ITGA2 (25,972-26,081 ; 0)
JER/ZMA	20:28,067-29,177	1,109 (223)	28,255 (4.57)	PARP8 (28,299-28,496 ; 44)
JER/ZMA	20:21,648-22,143	494.6 (135)	21,758 (4.19)	ACTBL2 (21,798-21,800 ; 40)
JER/ZMA	20:22,690-23,008	318.7 (107)	22,767 (4.32)	LOC112443061 (22,781-22,781 ; 14)
ZMA/JER	02:5,002-5,344	342.5 (80)	5,006 (3.55)	IWS1 (5,012-5,062 ; 7)
ZMA/JER	12:35,363-39,777	4,414 (731)	37,844 (4.01)	LOC132346771 (37,569-37,575 ; 269)
ZMA/JER	12:34,871-35,327	455.8 (77)	35,222 (3.69)	LOC101902228 (35,266-35,314 ; 45)
ZMA/JER	17:63,890-64,781	890.9 (208)	63,895 (4.2)	ACACB (63,846-63,961 ; 0)

Table S9: Description of the regions harboring footprints of selection based on the $Rsb_{JER/ZMA}$ tests. Tests were carried unilaterally to provide insights into the origin of the signals. The two regions that overlap with those identified in Table 1 highlighted with a ⋆.

Category	$-\log_{10}(P)$ range	Molecules
Nervous System Development and Function	4.5;1.4	ADGRG6,AGL,BLOC1S5,GCM1,MAPK10,SYNE2,USH1C,UTRN
Neurological Disease	4.5;1.3	ADGRG6,GCM1,HSPB3,MAPK10,OR8B8,PKHD1, SLC36A3,SYNE2,SYT16,TRPS1,USH1C,UTRN
Organismal Injury and Abnormalities	4.5;1.3	ADGRG6,AGL,BLOC1S5,BRINP2,GCM1,HSPB3,MAPK10,OR8B8, PKHD1,SLC36A3,STXBP6,SYNE2,SYT16,TRPS1,USH1C,UTRN
Cell Morphology	4.2;1.4	ADGRG6,AGL,MAPK10,PKHD1,SYNE2,TRPS1,USH1C,UTRN
Tissue Morphology	4.2;1.4	ADGRG6,AGL,MAPK10,PKHD1,SYNE2,TRPS1,USH1C,UTRN
Cancer	3.7;1.3	ADGRG6,AGL,BLOC1S5,BRINP2,GCM1,HSPB3,MAPK10,OR8B8, PKHD1,SLC36A3,STXBP6,SYNE2,SYT16,TRPS1,USH1C,UTRN
Gastrointestinal Disease	3.7;1.3	AGL,BRINP2,HSPB3,MAPK10,OR8B8, PKHD1,SLC36A3,STXBP6,SYNE2,SYT16,TRPS1,USH1C,UTRN
Respiratory Disease	3.7;1.3	ADGRG6,BRINP2,GCM1,MAPK10,OR8B8, PKHD1,STXBP6,SYNE2,SYT16,TRPS1,USH1C,UTRN
Cell-To-Cell Signaling and Interaction	3.4;1.3	GCM1,MAPK10,PKHD1,STXBP6,SYNE2,USH1C,UTRN
Cellular Assembly and Organization	3.4;1.3	ADGRG6,BLOC1S5,GCM1,MAPK10,PKHD1,SYNE2,USH1C,UTRN
Connective Tissue Disorders	3.2;1.3	ADGRG6,AGL,GCM1,TRPS1,UTRN
Skeletal and Muscular Disorders	3.2;1.3	ADGRG6,AGL,GCM1,HSPB3,STXBP6,SYNE2,TRPS1,UTRN
Auditory Disease	3.2;1.5	USH1C
Cardiovascular System Development and Function	3.2;1.3	PKHD1,SYNE2,UTRN
Cell Death and Survival	3.2;1.4	MAPK10,TRPS1,UTRN
Dermatological Diseases and Conditions	3.2;1.4	ADGRG6,BRINP2,MAPK10,OR8B8,PKHD1,SYNE2,SYT16,TRPS1,USH1C
Developmental Disorder	3.2;1.3	ADGRG6,AGL,BLOC1S5,GCM1,PKHD1,SYNE2,TRPS1,UTRN
Digestive System Development and Function	3.2;1.4	AGL,PKHD1,TRPS1,USH1C
Embryonic Development	3.2;1.5	BLOC1S5,GCM1,PKHD1,TRPS1,USH1C,UTRN
Hepatic System Disease	3.2;1.6	AGL,BRINP2,MAPK10,PKHD1,SYNE2
Hereditary Disorder	3.2;1.4	ADGRG6,AGL,BLOC1S5,HSPB3,MAPK10,PKHD1,SYNE2,TRPS1,USH1C,UTRN
Metabolic Disease	3.2;1.3	AGL,BLOC1S5,MAPK10,PKHD1,SLC36A3,SYT16,TRPS1,UTRN
Ophthalmic Disease	3.2;2	BLOC1S5,USH1C
Organ Development	3.2;1.5	BLOC1S5,PKHD1,TRPS1,USH1C,UTRN
Organismal Development	3.2;1.3	ADGRG6,AGL,BLOC1S5,GCM1,PKHD1,SYNE2,TRPS1,USH1C,UTRN
Skeletal and Muscular System Development and Function	3.2;1.5	ADGRG6,TRPS1,UTRN
Tissue Development	3.2;1.5	ADGRG6,BLOC1S5,GCM1,PKHD1,TRPS1,USH1C,UTRN
Auditory and Vestibular System Development and Function	2.9;1.7	BLOC1S5,USH1C
Behavior	2.9;1.5	TRPS1,USH1C
Carbohydrate Metabolism	2.9;1.6	AGL
Organ Morphology	2.9;1.3	AGL,BLOC1S5,PKHD1,SYNE2,TRPS1,USH1C,UTRN
Renal and Urological Disease	2.9;1.3	AGL,PKHD1,SYNE2,SYT16,TRPS1,USH1C,UTRN
Cellular Function and Maintenance	2.7;1.4	ADGRG6,BLOC1S5,PKHD1,STXBP6,SYNE2,USH1C,UTRN
Cellular Movement	2.7;1.6	BLOC1S5,MAPK10,SYNE2,UTRN
Endocrine System Disorders	2.7;1.3	AGL,BRINP2,MAPK10,PKHD1,SLC36A3,SYNE2,SYT16,TRPS1,UTRN
Lipid Metabolism	2.7;2.7	UTRN
Reproductive System Disease	2.7;1.3	ADGRG6,AGL,BLOC1S5,BRINP2,HSPB3,MAPK10,OR8B8,PKHD1, STXBP6,SYNE2,SYT16,TRPS1,USH1C,UTRN
Small Molecule Biochemistry	2.7;1.7	ADGRG6,MAPK10,PKHD1,STXBP6,UTRN
Hepatic System Development and Function	2.6;2.5	AGL,PKHD1
Hematological Disease	2.6;1.4	AGL,BRINP2,PKHD1,SLC36A3,STXBP6,SYNE2,SYT16,TRPS1
Immunological Disease	2.6;1.6	AGL,BRINP2,PKHD1,SLC36A3,STXBP6,SYNE2,SYT16,TRPS1
Cell Signaling	2.6;2	ADGRG6,MAPK10,TRPS1
Cellular Development	2.6;1.5	ADGRG6,GCM1,PKHD1,TRPS1,USH1C,UTRN
Cellular Growth and Proliferation	2.6;1.5	ADGRG6,PKHD1,TRPS1,UTRN
Connective Tissue Development and Function	2.6;1.5	PKHD1,TRPS1
Post-Translational Modification	2.6;1.6	HSPB3,MAPK10,UTRN
Cellular Compromise	2.5;2.0	MAPK10
Reproductive System Development and Function	2.5;2.5	GCM1
Protein Synthesis	2.3;1.5	AGL,OFCC1
Cell-mediated Immune Response	2.3;2.3	MAPK10
Hematological System Development and Function	2.3;1.7	MAPK10,PKHD1,UTRN
Immune Cell Trafficking	2.3;1.9	MAPK10,UTRN
Inflammatory Response	2.3;1.7	MAPK10,UTRN
Inflammatory Disease	2.2;2.2	UTRN
Drug Metabolism	2.1;1.9	MAPK10,STXBP6
Molecular Transport	2.1;1.6	AGL,MAPK10,STXBP6
Nucleic Acid Metabolism	2.1;2.1	ADGRG6
Renal and Urological System Development and Function	2.1;2.0	PKHD1
Respiratory System Development and Function	2.0;1.3	TRPS1
Hair and Skin Development and Function	1.9;1.6	SYNE2,TRPS1
Amino Acid Metabolism	1.7;1.7	MAPK10
Organismal Survival	1.7;1.5	ADGRG6,AGL,GCM1,MAPK10,SYNE2,TRPS1,UTRN
Cardiovascular Disease	1.7;1.3	SYNE2,UTRN
Psychological Disorders	1.7;1.7	MAPK10
Tumor Morphology	1.6;1.6	MAPK10
Visual System Development and Function	1.5;1.5	USH1C
Cell Cycle	1.4;1.4	PKHD1
DNA Replication, Recombination, and Repair	1.4;1.4	PKHD1

Table S10: List of significant diseases and functions associated with the 17 candidate genes detected under selection in the cattle population from Amsterdam island. Results were generated with Ingenuity Pathway Analysis (Ingenuity Systems, Inc., 2023)

Ingenuity Canonical Pathways	$-\log_{10}(P)$	Molecules
EGR2 and SOX10-mediated initiation of Schwann cell myelination	3.73	ADGRG6,UTRN
Agrin Interactions at Neuromuscular Junction	2.97	MAPK10,UTRN
IL-6 Signaling	2.43	HSPB3,MAPK10
SNARE Signaling Pathway	2.39	STXBP6,SYT16
Glycogen Degradation II	2.11	AGL
Glycogen Degradation III	2.04	AGL
Docosahexaenoic Acid (DHA) Signaling	1.88	STXBP6,SYT16
Oxytocin Signaling Pathway	1.78	HSPB3,MAPK10
Glycogen metabolism	1.78	AGL
IL-22 Signaling	1.78	MAPK10
IL-17A Signaling in Gastric Cells	1.74	MAPK10
Apelin Liver Signaling Pathway	1.73	MAPK10
Synaptogenesis Signaling Pathway	1.69	STXBP6,SYT16
MAPK targets/ Nuclear events mediated by MAP kinases	1.64	MAPK10
4-1BB Signaling in T Lymphocytes	1.63	MAPK10
Inhibition of Angiogenesis by TSP1	1.63	MAPK10
MAP kinase activation	1.57	MAPK10
April Mediated Signaling	1.54	MAPK10
B Cell Activating Factor Signaling	1.53	MAPK10
MIF Regulation of Innate Immunity	1.52	MAPK10
Apelin Pancreas Signaling Pathway	1.49	MAPK10
UVC-Induced MAPK Signaling	1.45	MAPK10
UVB-Induced MAPK Signaling	1.43	MAPK10
Meiotic synapsis	1.42	SYNE2
Sensory processing of sound by outer hair cells of the cochlea	1.42	USH1C
CD27 Signaling in Lymphocytes	1.41	MAPK10
Role of IL-17A in Arthritis	1.39	MAPK10
GADD45 Signaling	1.38	MAPK10
PCP (Planar Cell Polarity) Pathway	1.38	MAPK10
Activation of IRF by Cytosolic Pattern Recognition Receptors	1.35	MAPK10
Induction of Apoptosis by HIV1	1.35	MAPK10
Sensory processing of sound by inner hair cells of the cochlea	1.32	USH1C
CD40 Signaling	1.32	MAPK10
IL-17A Signaling in Airway Cells	1.32	MAPK10

Table S11: List of significant canonical pathways associated with the 17 candidate genes detected under selection in the cattle population from Amsterdam Island. Results were generated with Ingenuity Pathway Analysis (Ingenuity Systems, Inc., 2023)

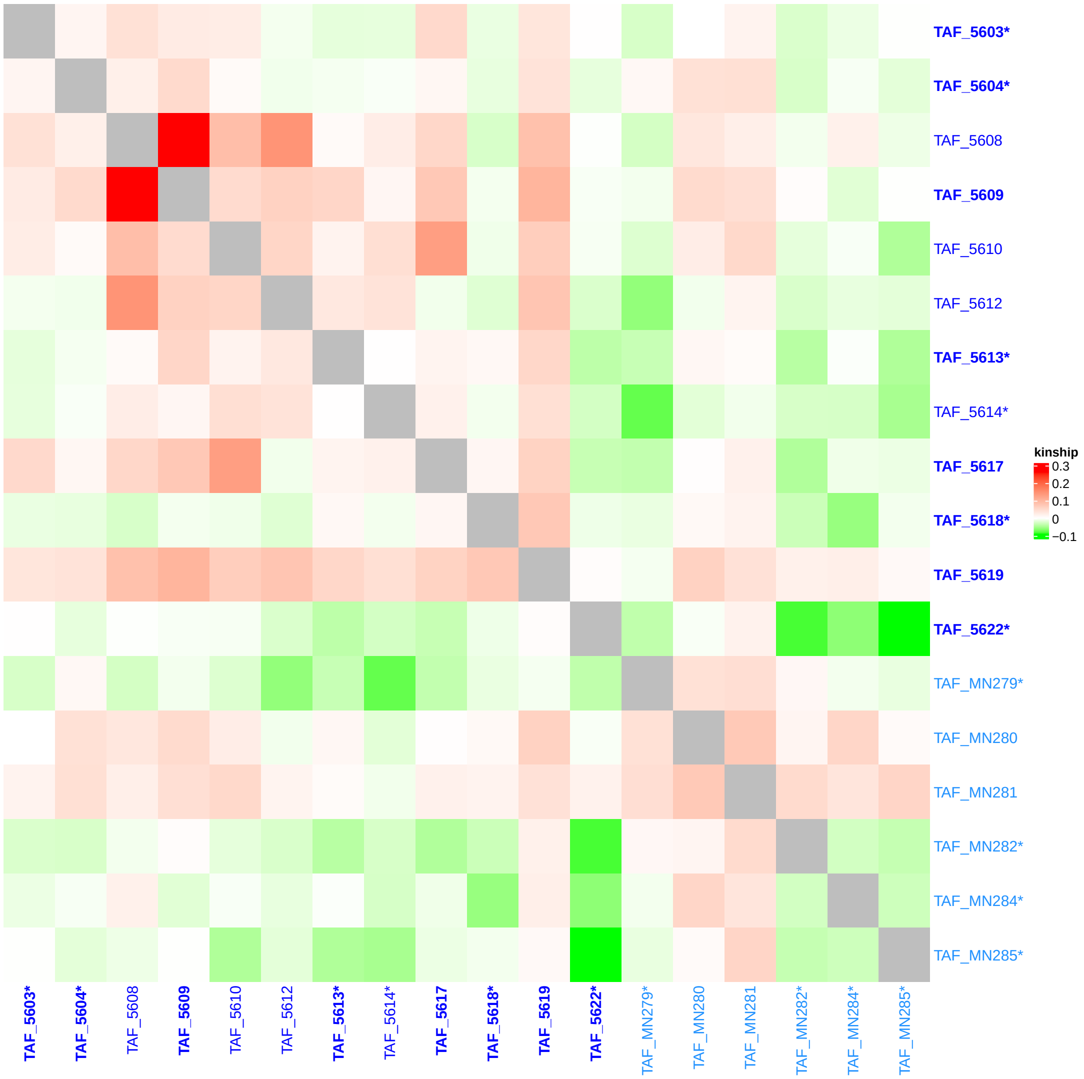

Figure S1: Heatmap of the kinship matrix among the 18 TAF individuals estimated with `King` (Manichaikul *et al.*, 2010). The names of the 12 (resp. 6) individuals collected in 1992 (resp. 2006) are colored in blue (resp. light blue). The eight WGS individuals (all sampled in 1992) are highlighted in bold, and the names of the ten individuals deemed unrelated (up to the second degree) followed by an asterisk (*) for identification. Among the 66 pairs of 1992 samples, the estimated kinship coefficients ranged from -0.039 to 0.26 (median of 0.014). The one between TAF_5609 (born in 1985) and TAF_5608 (born in 1989) was within $[0.177, 0.354]$, suggesting a first degree relationship inferred to be a parent/offspring relationship from IBD-segment sharing (here mother/daughter from the birthdates and sex). The four coefficients between TAF_5608 and TAF_5612 ($\phi = 0.144$); TAF_5610 and TAF_5617 ($\phi = 0.131$); TAF_5609 and TAF_5619 ($\phi = 0.101$) and TAF_5608 and TAF_5610 ($\phi = 0.0887$) lie within $[0.0884, 0.177]$ suggesting a second degree relationship. Analysis of IBD-segment sharing allowed upgrading the relationship between TAF_5608 and TAF_5612 to a full-sib relationship, birthdates suggesting half-sibs or aunt-niece relationships for the three other ones. The 61 remaining coefficients between the 1992 individuals were all < 0.0884 (relationship more distant than second-degree). Likewise, among the 15 pairs of 2006 samples, the estimated kinship coefficients were < 0.0884, ranging from -0.035 to 0.074 (median of 0.013); as the 72 pairwise comparisons between individuals collected in 1992 and 2006 which ranged from -0.091 to 0.062 (median of -0.0081).

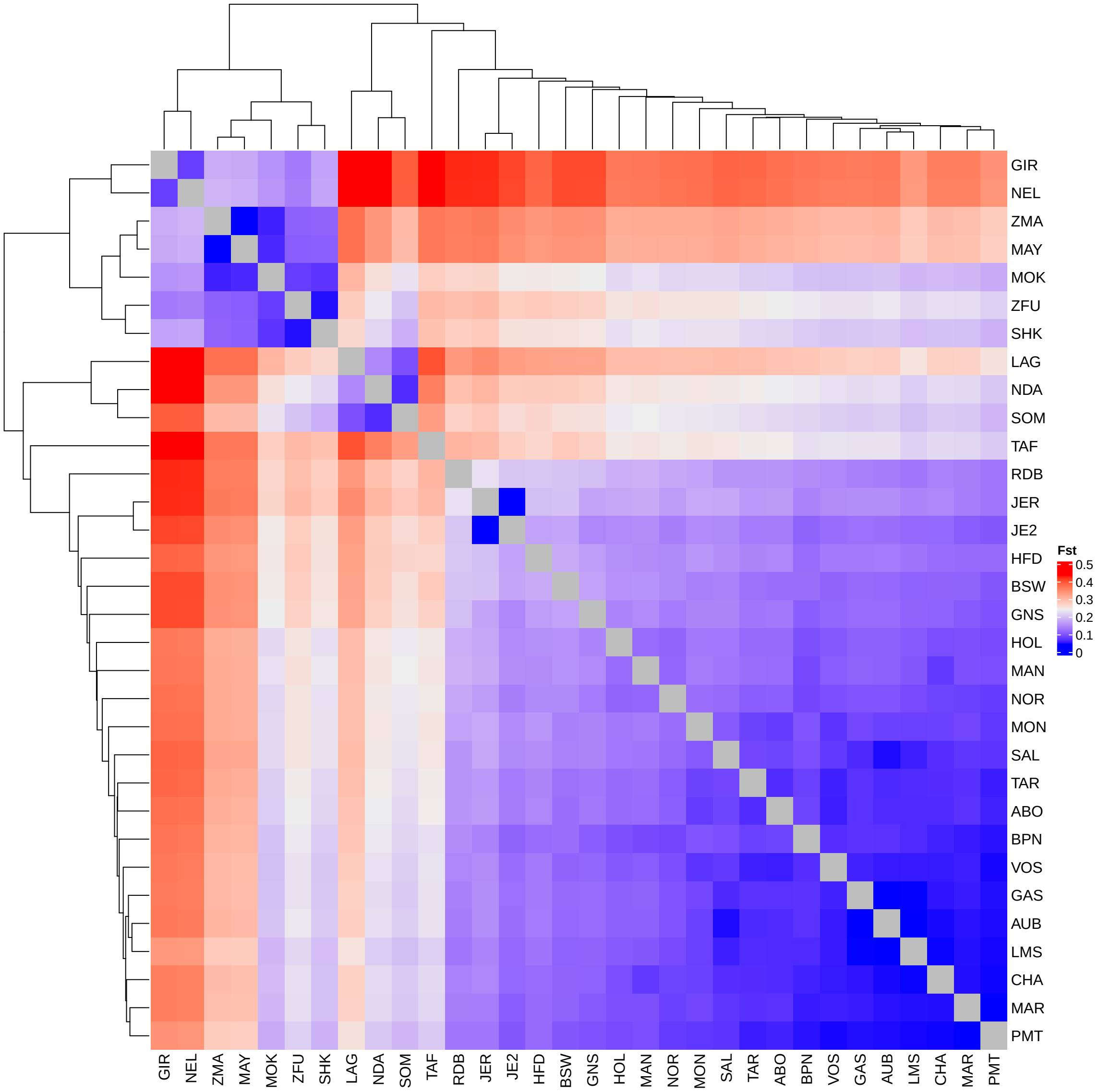

Figure S2: Heatmap of the pairwise-population F_{ST} values between all the 32 populations estimated from genotyping data on the 40,426 autosomal SNPs. The estimated pairwise F_{ST} values range from 0.0247 for the MAY/ZMA pair, up to 0.472 for the LAG/NEL pair, with a median of 0.203. For pairs including newly genotyped sample MOK, F_{ST} values range from 0.0578 (MOK/ZMA) to 0.303 (MOK/LAG). For pairs including TAF, values range from 0.208 (TAF/PMT) to 0.436 (TAF/NEL). The TAF/PMT pairs have the lowest pairwise-F_{ST} compared to others, likely because the PMT (EUT) breed has a small amount of ZEB ancestry, similar to the TAF (see Figure S3 and (Gautier *et al.*, 2010).

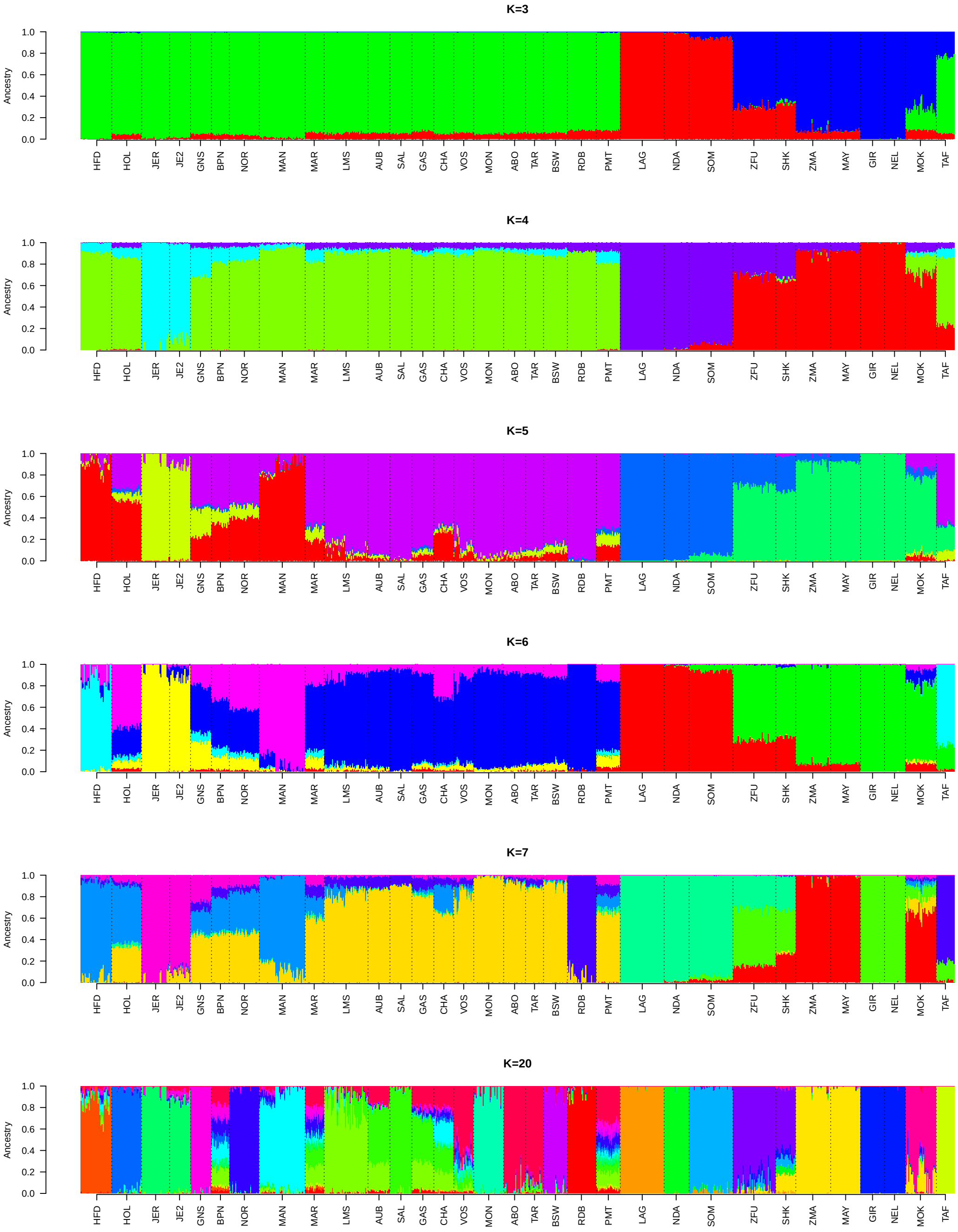

Figure S3: Unsupervised hierarchical clustering of the 876 individuals from the 32 populations based on genotyping data at 40,426 autosomal SNPs into K=3 to K=7 and K=20 (the most suitable value according to cross-validation analyses) clusters.

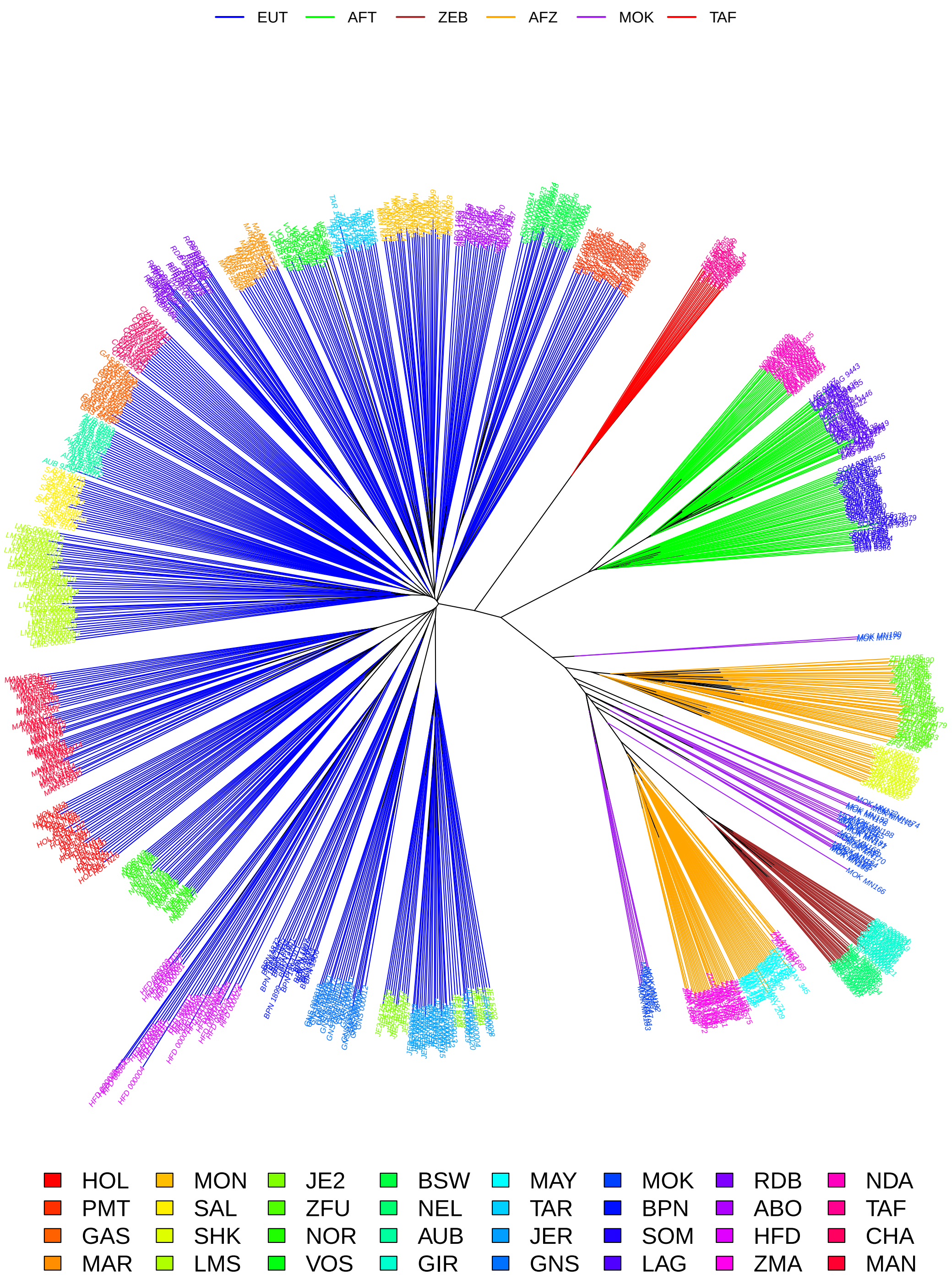

Figure S4: Neighbor-Joining tree relating the 876 individuals from the 32 populations based on allele-sharing distance computed on the 40,426 autosomal SNPs. Edges are colored according to the population group (top legend) and tip individual names according to the population of origin (bottom legend).

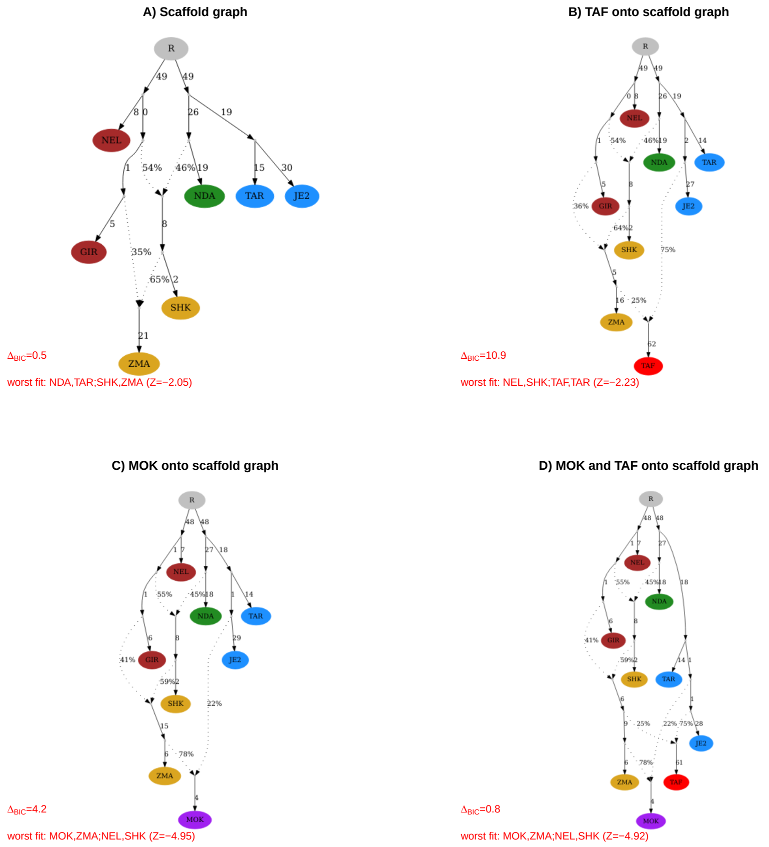

Figure S5: Admixture graph construction based on the W50K dataset with the R package `poolfstat` (Gautier *et al.*, 2022). A tree consisting of two ZEB (GIR and NEL), one AFT (NDA) and two distantly related EUT breeds (JE2 and TAR) was first used as a scaffold to which the SHK (East-African AFZ) and ZMA were jointly added to represent the history of Indian Ocean Zebus (Magnier *et al.*, 2022). The resulting best fitting graph is plotted in A) and was in agreement with results obtained by (Magnier *et al.*, 2022) using BovineHD data. Yet, the positioning of the ancestral source population of SHK that is related to the ZEB ancestor of GIR and NEL (i.e., either on the branch leading to GIR as in A) or on the branch leading to NEL or ancestral to ZEB) lead to graph with closely related BIC. B) Best fitting admixture graph among all possible ways of positioning TAF onto the scaffold graph obtained in A. This graph showing that the TAF population has an admixed origin with two sources related to JE2 ($\alpha = 75\%$) and ZMA ($1 - \alpha = 25\%$) had a very high support (BIC 10.9 units lower than the graph with the second lowest BIC) and good fit to the observed f-statistics (the worst fitted f-stats associated $|Z| = 2.23$). C) Best fitting admixture graph among all possible ways of positioning MOK onto the scaffold graph obtained in A. This graph showing that the MOK population has an admixed origin with two sources, JE2 ($\alpha = 22\%$) and ZMA ($1 - \alpha = 78\%$), had good support (BIC 4.2 units lower than the graph with the second lowest BIC) but less optimal fit to the observed f-statistics (the worst fitted f-stats associated $|Z| = 4.95$). Note that the worst fitted f-statistics ($f_4(MOK, ZMA; NEL, SHK)$) suggests that this graph fails to capture some direct ZEB (represented by NEL) ancestry to the MOK. Such ancestry would indeed contribute positively to $f_4(MOK, ZMA; NEL, SHK)$ making the observed f-statistics higher than the fitted f-statistics (and the associated Z-score negative). Such a three-way admixture origin (EUTxZMAxZEB) of the MOK would be consistent with historical record. D) Best fitting admixture graph among all possible ways of jointly positioning TAF and MOK onto the scaffold graph obtained in A. This graph is in agreement with B) and C) and suggests that MOK might not be viewed as the closest proxy to the source populations from which TAF originates although MOK and TAF share closely related ancestral EUT sources (the relative positioning of which led to graphs with close BIC).

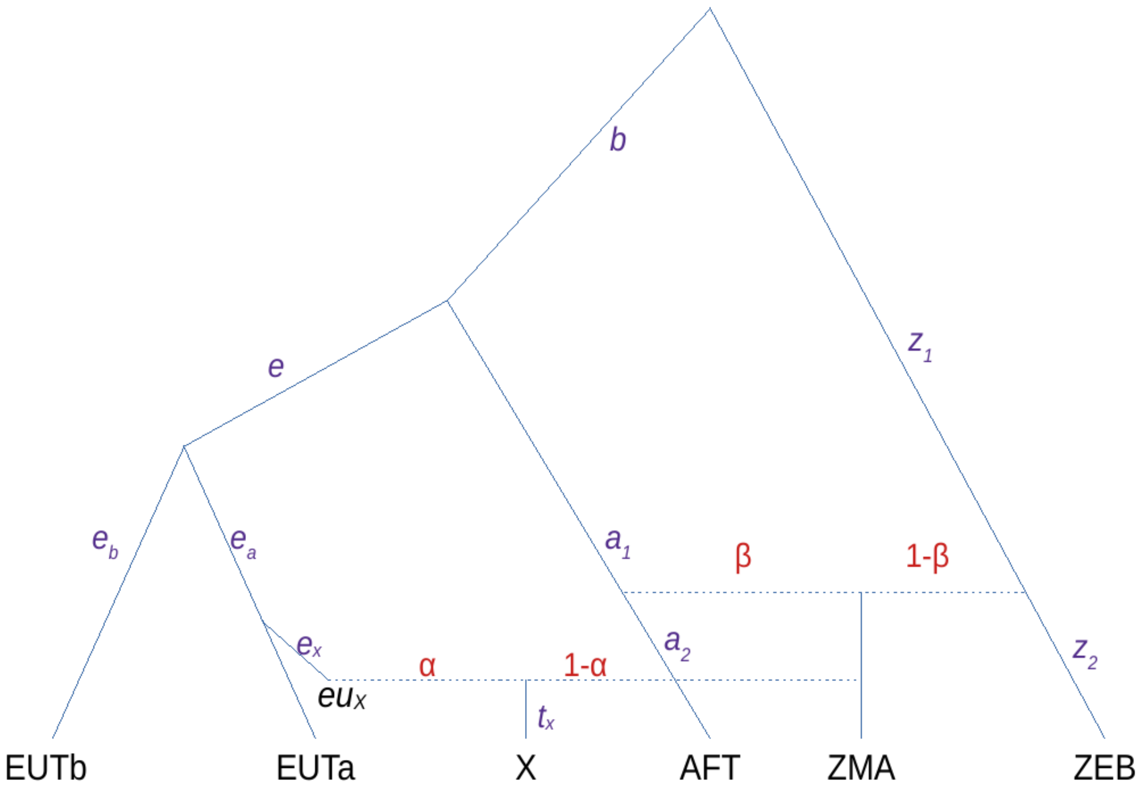

Figure S6: Schematic representation of the expected F_4 for various configuration under the (simplified) scenario associated to the inferred admixture graph describing the origin of an admixed population X (e.g., X=TAF or X=MOK) with one source related to one EUT and the other to the ZMA which is itself admixed. Note the ZEBxAFT admixed origin of one of the two ZMA sources can be disregarded for our configurations of interest. Under this scenario:

$$F_3(X;ZMA,EUT_b) = \tau_X - \beta(1-\beta)\Big((1-\alpha)^2(z_1+b) + \alpha^2 a_1 + a_2 + e\Big) = \gamma$$

$$F_3(X;ZMA,EUT_a) = \gamma - \beta(1-\beta)e_a$$

Hence, the EUT population EUT_a, the closest to the European source of X (eu_X) is expected to display the lowest f_3 value for the $(X;ZMA,EUT)$ configurations. Likewise,

$$F_4(EUT_b,AFT;X,ZEB) = (\alpha e - (1-\alpha)\beta a_1) = \gamma$$

$$F_4(EUT_a,AFT;X,ZEB) = \alpha e_a + \gamma$$

Hence, the EUT population EUT_a, the closest to the European source of X (eu_X) is expected to display the highest f_4 value for the $(EUT,AFT;X,ZEB)$ configurations. In addition,

$$F_4(EUT_b,ZEB;ZMA,EUTa) = (z_1+b)(\beta-1) - e = \tau$$

$$F_4(EUT_b,ZEB;ZMA,X) = \alpha\tau$$

The F_4-ratio= $\frac{F_4(EUT_b,ZEB;ZMA,X)}{F_4(EUT_b,ZEB;ZMA,EUTa)}$ then provides an estimator of the European source admixture rate α.

A)

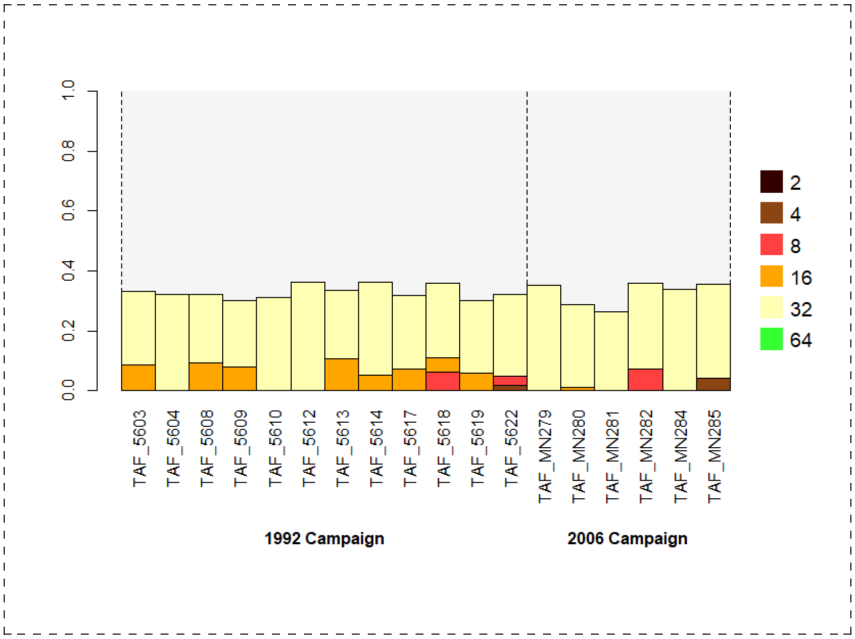

B)

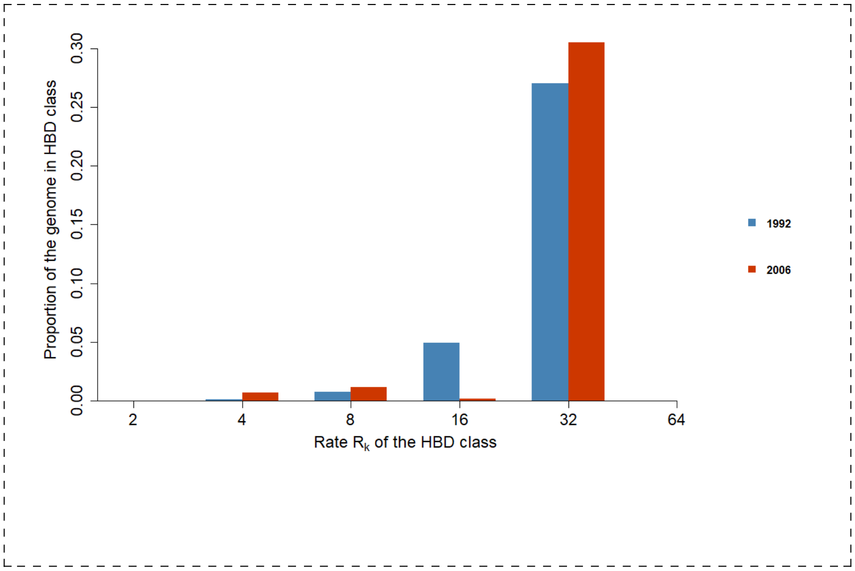

Figure S7: Partitioning of inbreeding levels in all TAF individuals using 50K SNP genotyping data (W50K dataset). A) Partitioning of the inbreeding levels into 6 different HBD classes for each individual separated by sampling campaign. B) Overall distribution of inbreeding levels among all individuals for the two sampling campaign (1992 and 2006).

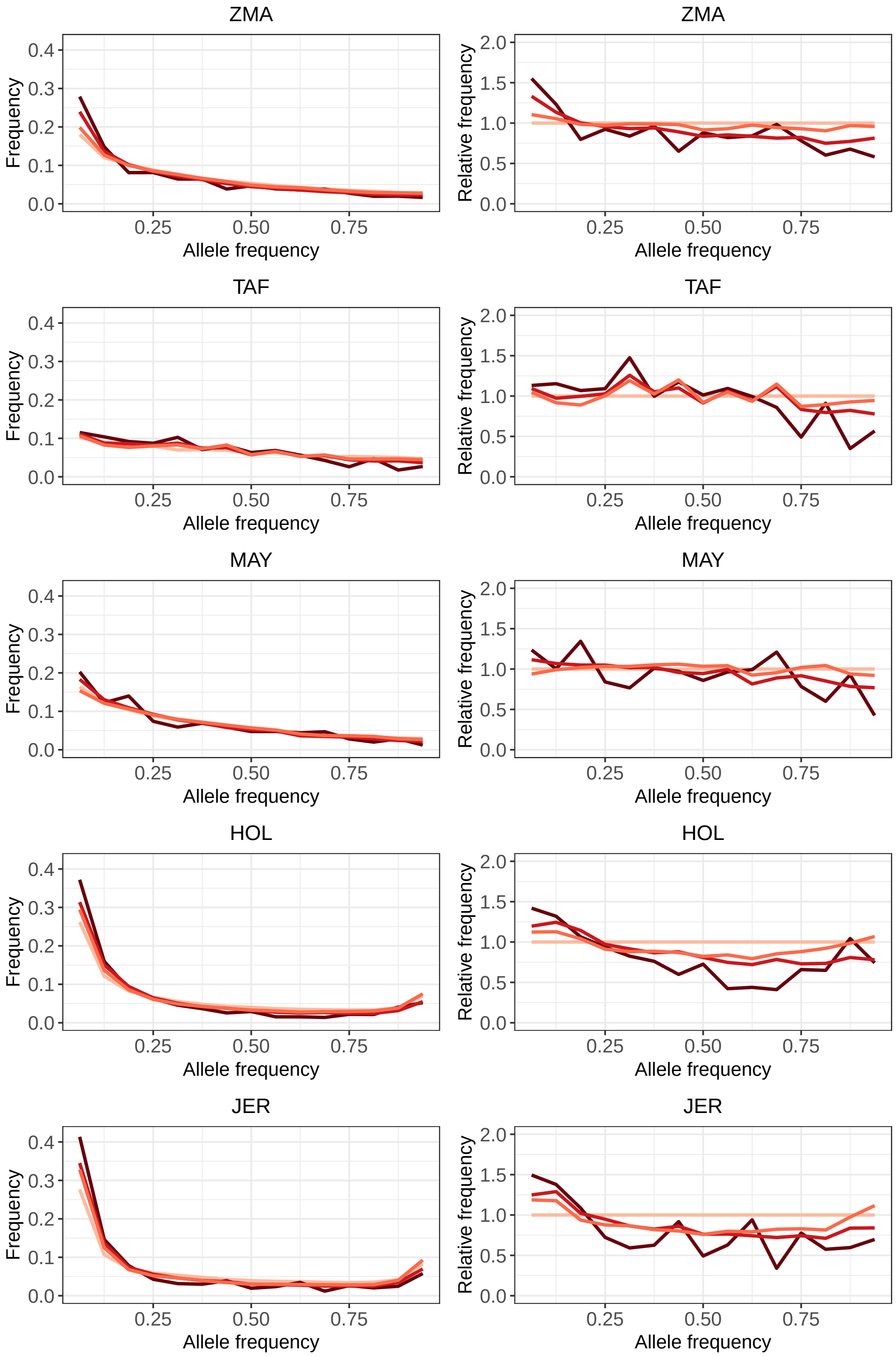

Figure S8: Comparison of the Site Frequency Spectrum (SFS) for intergenic (IG), Synonymous (S), Non-Synonymous (NS) and Loss-of-Function (LoF) variants in the five breeds colored from light to darkred as in Figure 4. The relative values were estimated by dividing with the frequencies estimated for intergenic variants.

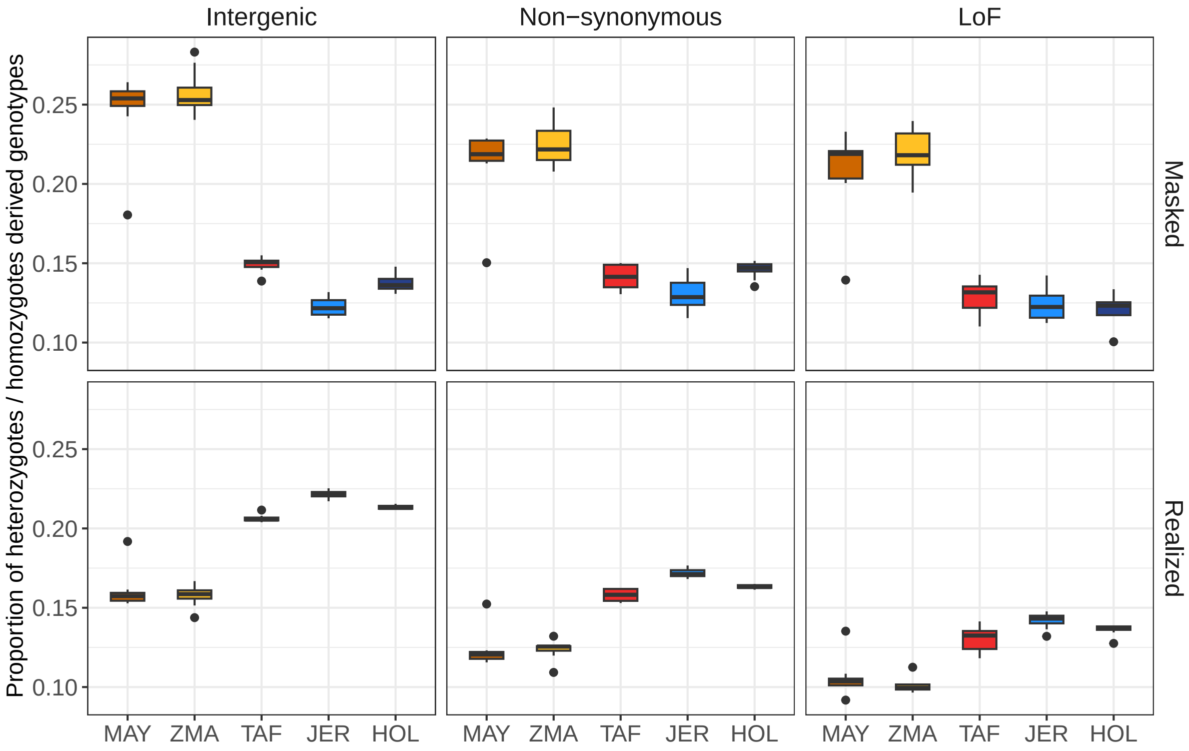

Figure S9: Distribution of the individual masked and realized loads within each population. The load were estimated per individual for intergenic, non-synonymous and loss-of-function (LoF) variants.

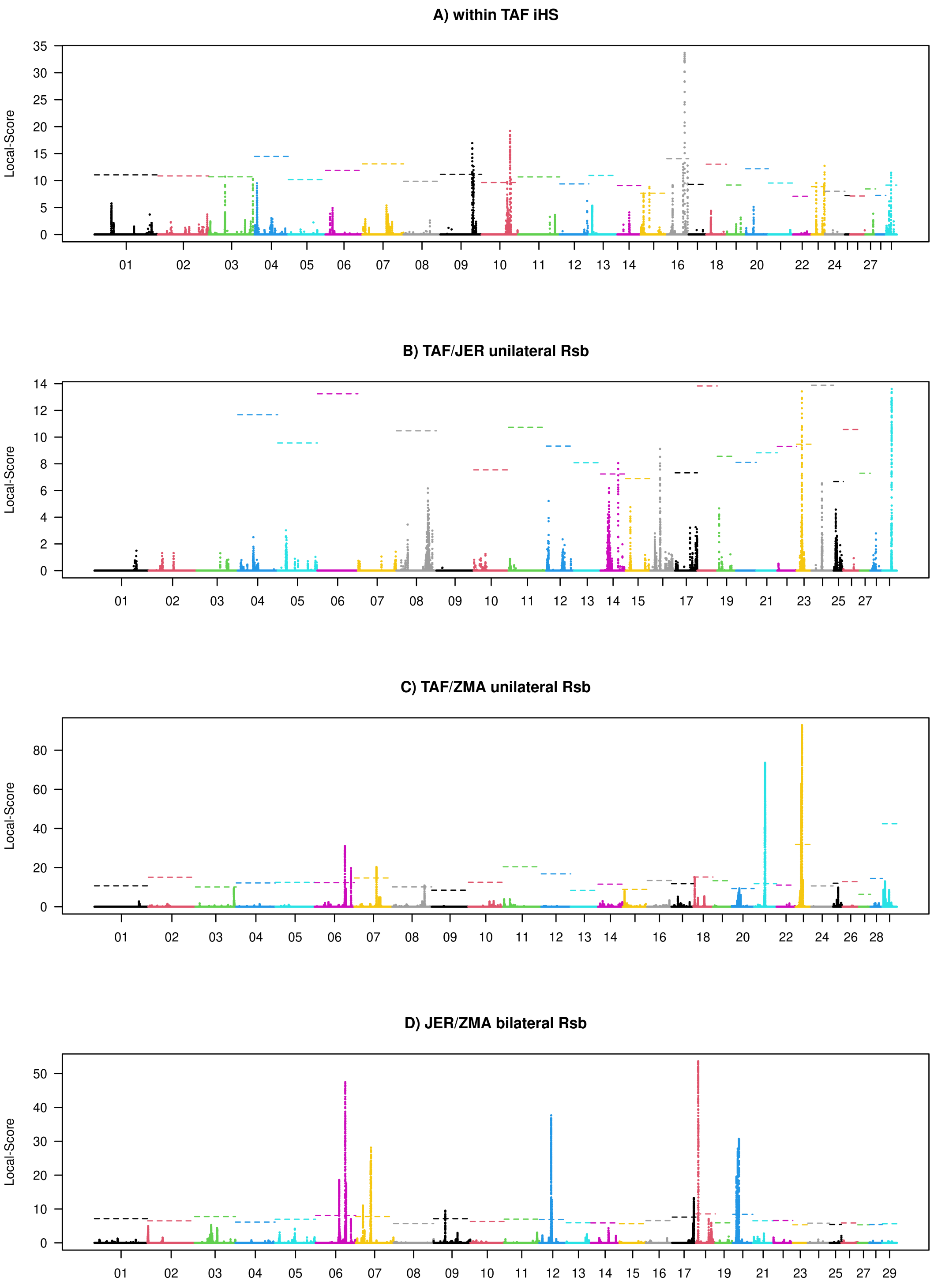

Figure S10: Manhattan plot over the bovine genome of the local scores derived from the within TAF *iHS* and the three pairwise *Rsb* statistics used to identify footprints of selection. The horizontal dashed lines indicate the chromosome specific 1% P-value threshold on the local score that was apply to select the significant windows.

References

Daetwyler, H. D., Capitan, A., Pausch, H., Stothard, P., Van Binsbergen, R., Brøndum, R. F., Liao, X., Djari, A., Rodriguez, S. C., Grohs, C., *et al.* 2014. Whole-genome sequencing of 234 bulls facilitates mapping of monogenic and complex traits in cattle. *Nature genetics*, 46(8): 858–865.

Flori, L., Moazami-Goudarzi, K., Alary, V., Araba, A., Boujenane, I., Boushaba, N., Casabianca, F., Casu, S., Ciampolini, R., Coeur D'Acier, A., *et al.* 2019. A genomic map of climate adaptation in mediterranean cattle breeds. *Molecular ecology*, 28(5): 1009–1029.

Gautier, M., Flori, L., Riebler, A., Jaffrézic, F., Laloé, D., Gut, I., Moazami-Goudarzi, K., and Foulley, J.-L. 2009. A whole genome bayesian scan for adaptive genetic divergence in west african cattle. *BMC genomics*, 10(1): 1–18.

Gautier, M., Laloë, D., and Moazami-Goudarzi, K. 2010. Insights into the genetic history of french cattle from dense snp data on 47 worldwide breeds. *PloS one*, 5(9): e13038.

Gautier, M., Vitalis, R., Flori, L., and Estoup, A. 2022. f-Statistics estimation and admixture graph construction with Pool-Seq or allele count data using the R package poolfstat. *Molecular Ecology Resources*, 22(4): 1394–1416.

Ingenuity Systems, Inc. 2023. Ingenuity pathway analysis (ipa) software. `https://www.qiagenbioinformatics.com/products/ingenuity-pathway-analysis/`. Accessed on Sept. 2023.

Li, H., Handsaker, B., Wysoker, A., Fennell, T., Ruan, J., Homer, N., Marth, G., Abecasis, G., and Durbin, R. 2009. The sequence alignment/map format and samtools. *Bioinformatics*, 25(16): 2078–9.

Magnier, J., Druet, T., Naves, M., Ouvrard, M., Raoul, S., Janelle, J., Moazami-Goudarzi, K., Lesnoff, M., Tillard, E., Gautier, M., *et al.* 2022. The genetic history of mayotte and madagascar cattle breeds mirrors the complex pattern of human exchanges in western indian ocean. *G3*, 12(4): jkac029.

Manichaikul, A., Mychaleckyj, J. C., Rich, S. S., Daly, K., Sale, M., and Chen, W.-M. 2010. Robust relationship inference in genome-wide association studies. *Bioinformatics*, 26(22): 2867–2873.

Matukumalli, L. K., Lawley, C. T., Schnabel, R. D., Taylor, J. F., Allan, M. F., Heaton, M. P., O'Connell, J., Moore, S. S., Smith, T. P., Sonstegard, T. S., *et al.* 2009. Development and characterization of a high density snp genotyping assay for cattle. *PloS one*, 4(4): e5350.

Sempéré, G., Moazami-Goudarzi, K., Eggen, A., Laloë, D., Gautier, M., and Flori, L. 2015. Widde: a web-interfaced next generation database for genetic diversity exploration, with a first application in cattle. *BMC genomics*, 16(1): 1–8.

Wu, N., Zhang, S., Li, X., Cao, Y., Liu, X., Wang, Q., Liu, Q., Liu, H., Hu, X., Zhou, X. J., James, A. A., Zhang, Z., Huang, Y., and Zhan, S. 2019. Fall webworm genomes yield insights into rapid adaptation of invasive species. *Nature Ecology and Evolution*, 3(1): 105–115.
